# The *Staphylococcus aureus* Response to Unsaturated Long Chain Free Fatty Acids: Survival Mechanisms and Virulence Implications

**DOI:** 10.1371/journal.pone.0004344

**Published:** 2009-02-02

**Authors:** John G. Kenny, Deborah Ward, Elisabet Josefsson, Ing-Marie Jonsson, Jason Hinds, Huw H. Rees, Jodi A. Lindsay, Andrej Tarkowski, Malcolm J. Horsburgh

**Affiliations:** 1 School of Biological Sciences, University of Liverpool, Liverpool, United Kingdom; 2 Department of Rheumatology and Inflammation Research, University of Gothenburg, Göteborg, Sweden; 3 Division of Cellular & Molecular Medicine, St George's, University of London, London, United Kingdom; University of Minnesota, United States of America

## Abstract

*Staphylococcus aureus* is an important human commensal and opportunistic pathogen responsible for a wide range of infections. Long chain unsaturated free fatty acids represent a barrier to colonisation and infection by *S. aureus* and act as an antimicrobial component of the innate immune system where they are found on epithelial surfaces and in abscesses. Despite many contradictory reports, the precise anti-staphylococcal mode of action of free fatty acids remains undetermined. In this study, transcriptional (microarrays and qRT-PCR) and translational (proteomics) analyses were applied to ascertain the response of *S. aureus* to a range of free fatty acids. An increase in expression of the σ^B^ and CtsR stress response regulons was observed. This included increased expression of genes associated with staphyloxanthin synthesis, which has been linked to membrane stabilisation. Similarly, up-regulation of genes involved in capsule formation was recorded as were significant changes in the expression of genes associated with peptidoglycan synthesis and regulation. Overall, alterations were recorded predominantly in pathways involved in cellular energetics. In addition, sensitivity to linoleic acid of a range of defined (*sigB, arcA, sasF, sarA, agr, crtM*) and transposon-derived mutants (*vraE, SAR2632*) was determined. Taken together, these data indicate a common mode of action for long chain unsaturated fatty acids that involves disruption of the cell membrane, leading to interference with energy production within the bacterial cell. Contrary to data reported for other strains, the clinically important EMRSA-16 strain MRSA252 used in this study showed an increase in expression of the important virulence regulator RNAIII following all of the treatment conditions tested. An adaptive response by *S. aureus* of reducing cell surface hydrophobicity was also observed. Two fatty acid sensitive mutants created during this study were also shown to diplay altered pathogenesis as assessed by a murine arthritis model. Differences in the prevalence and clinical importance of *S. aureus* strains might partly be explained by their responses to antimicrobial fatty acids.

## Introduction

*Staphylococcus aureus* is the aetiological agent for a wide range of human infections, including abscesses, septicaemia, arthritis and endocarditis. The increased prevalence of meticillin resistant- (MRSA) and vancomycin insensitive-*S. aureus* strains, and the emergence of community-acquired MRSA make investigations into the pathogenicity of this species imperative. Inevitably, this focuses research into the development of novel antimicrobial agents, which requires a rigorous study of staphylococcal physiology. Long chain unsaturated free fatty acids (LC-uFFAs), typically ≥C16, are known to possess anti-staphylococcal activity and LC-uFFAs are important components of the innate immune system. Individuals with atopic dermatitis exhibit deficient production of the skin-specific LC-uFFA, hexadecenoic acid [C16:1 (n-6)], which is associated with increased carriage of *S. aureus* and susceptibility to bacterial skin infections [1]–[3]. In human tissue and nasal fluid, the major LC-FFAs are the unsaturated linoleic [C18:2 (n-6,9)], oleic [C18:1 (n-9)] and palmitoleic [C16:1 (n-7)] acids and the saturated palmitic [C16:0] and stearic [C18:0] acids [4]–[7]. Assay of staphylococcal abscess homogenates has revealed the presence of anti-staphylococcal activity comprising a pool of monoglycerides and free fatty acids [8]–[10]. The most abundant compound present in this active pool was identified as linoleic acid and was found at millimolar concentrations.

FFAs of various chain lengths and with different levels of unsaturation are primarily effective against Gram-positive bacteria [11]–[18]. Inhibition of several membrane-enveloped viruses has also been demonstrated [19]–[21]. Although several studies have attempted to pinpoint the specific cellular target(s) of LC-uFFAs, the actual anti-bacterial mechanism has not been unambiguously determined. Conflicting data have proposed that LC-uFFAs inhibit all major bacterial biosynthetic pathways within the cell, or alternatively, that they specifically inhibit FabI, which catalyses the final and rate-limiting step in fatty acid biosynthesis [12], [18], [22], [23]. Oleic acid was proposed by Won *et al*. [24] to inhibit glucosyltransferases, while other proposed mechanisms for LC-uFFA-mediated growth inhibition include peptidoglycan (PG) precipitation, peroxidative stress, interference with energy metabolism and alteration of the membrane permeability or fluidity [12], [16], [18], [22], [25], [26].

A diversity of mechanisms have been proposed to account for resistance to LC-uFFAs in *S. aureus*. Enhanced production of the carotenoid staphyloxanthin (giving *aureus* its golden title) has been proposed as a mechanism to relieve the inhibitory effects of increased membrane fluidity due to insertion of LC-uFFAs into the lipid bilayer in *S. aureus* [26]–[28]. Increased staphylococcal resistance to LC-uFFAs was positively correlated with pigmentation, although these experiments were performed using non-isogenic strains [28]. A fatty acid modifying enzyme (FAME), which catalyses the esterification of FFAs with cholesterol has also been purified from several *S. aureus* strains and its production correlated with increased disease severity in an abscess model [29]–[32]. Nonetheless the gene encoding FAME remains unidentified. Furthermore, in *Neisseria gonorrhoea*, FFA resistance has been linked to the presence of FFA-specific efflux pumps [33] while in *S. aureus*, the expression of Fur-iron-regulated staphylococcal surface-associated protein IsdA was identified as contributing to FA resistance in iron-limited environments by reducing cellular hydrophobicity [34]. Another proposed mechanism included the increased production of a ‘protective slime’ composed of precipitated PG complexed to fatty acids [25].

Previous studies demonstrated that *S. aureus* responds to the C12 monoester glycerol monolaurate (GML) and the component FFA lauric acid by reducing levels of expression of alpha toxin (Hla) [35]–[37]. Similarly, Clarke *et al*. [34] showed that expression of *hla* was reduced following exposure of *S. aureus* to the LC-uFFA hexadecenoic acid [C16:1 (n-6)]. More recently, GML was shown to inhibit the synthesis of toxins in several Gram-positive bacteria and also limited the effect of these toxins on eukaryotic cells [38]–[40].

While the biological effects of free fatty acids as antimicrobial compounds have been catalogued, there remains no unequivocal identification of the targets or mechanisms of action in relation to *S. aureus*. Transcriptomic and proteomic analyses have the potential to elucidate complex cellular and metabolic responses and are applied here for the first time to analyse the reaction of *S. aureus* to the LC-uFFAs linoleic, oleic and hexadecenoic acid. In addition, an analysis of existing well-characterised mutants and the generation of new allelic replacement mutants based on gene array data coupled to transposon screens was carried out to identify loci important for survival. Finally, a murine arthritis model of infection was used to ascertain whether two of the genes highlighted in this study have a role in pathogenesis.

## Results

### Comparative resistance of *S. aureus* strains to unsaturated C18 free fatty acids

**Figure 1:**
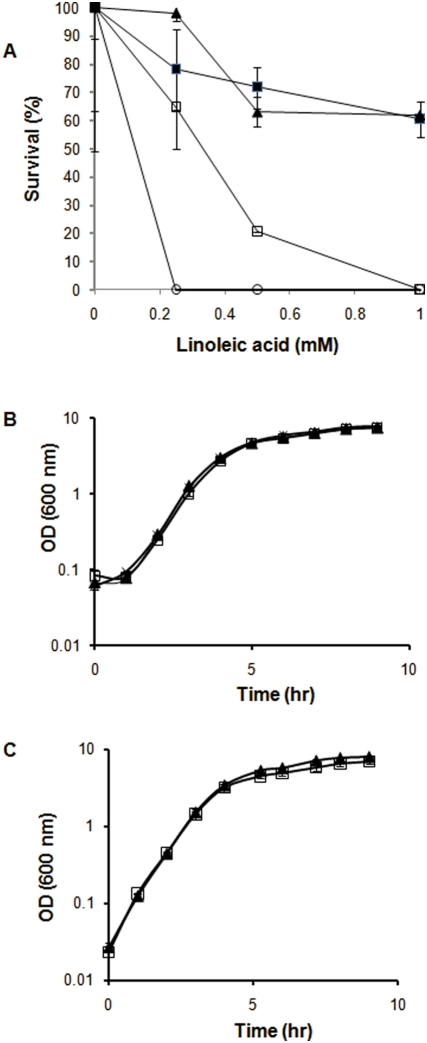
Inhibition of *S. aureus* by C18 unsaturated fatty acids. A Graph showing percentage survival of wild-type strains of *S. aureus* when these strains were incubated on BHI plates containing 0, 0.25, 0.5 and 1 mM linoleic acid. The strains analysed were SH1000 (closed box), MRSA252 (closed triangle), MSSA476 (open box) and N315 (open circle). This assay was performed in triplicate and is representative of multiple experiments. B Growth of a 0.5% (vol/vol) inoculum of MRSA252 in 100 ml BHI containing 0 mM fatty acid (closed triangle), 0.01 mM oleic acid (cross) or 0.01 mM linoleic acid (open box) at 37°C with shaking at 250 rpm. RNA was extracted from these cells at an OD_600_ of 3 and analysed in microarray experiments as the growth exposure conditions. C Growth of a 0.5% (vol/vol) inoculum of MRSA252 in 100 ml BHI at 37°C with shaking at 250 rpm with (open box) or without (closed triangle) the addition of 0.1 mM linoleic acid at an OD_600_ of 3. RNA was extracted from these cells 20 min post-exposure and analysed in microarray experiments as the challenge conditions. The growth curves shown in B and C were performed in biological triplicate. The error bars shown in graphs B and C correspond to standard errors of the mean.

The relative resistances of different strains of *S. aureus* to the unsaturated C18 free fatty acids linoleic acid [C18:2 (n-6,9)] and oleic acid [C18:1 (n-9)] were compared using a previously described agar plate assay [13]. Many strains, such as MSSA476 and N315, were unable to grow on emulsion agar plates containing 1 mM linoleic acid (Fig. 1A). In contrast MRSA252, an epidemic ERMSA-16 strain, and the laboratory strain SH1000 displayed high levels (>60%) of survival at millimolar concentrations. Consequently, all subsequent experiments were performed using MRSA252 and SH1000 strains of *S. aureus,* owing to their enhanced growth in the presence of C18 LC-uFFAs.

### Growth of MRSA252 in the presence of LC-uFFAs

To facilitate analysis of gene transcription and protein expression, a range of different concentrations of linoleic or oleic acid and the timing of their addition were examined during growth (data not shown). Upon inoculation 0.01 mM linoleic acid was determined to be the maximum concentration, which did not retard the aerobic growth of MRSA252 in BHI broth (Fig. 1B). Cells were subsequently grown in the presence of 0.01 mM linoleic or oleic acid with the FFAs being added at the start of growth (growth exposure conditions). To test the response of MRSA252 to LC-uFFAs under slightly different conditions, a higher concentration of linoleic acid (0.1 mM) was added during the late-exponential growth phase (OD_600_ = 3) where it was observed to reduce subsequent growth (challenge conditions) (Fig. 1C). These culture conditions were repeated for independent samples and cells were harvested to determine the transcriptional and translational responses of the cells to treatment with LC-uFFAs.

### The transcriptional response of *S. aureus* to C18 free fatty acids

A pronounced differential transcriptional response was observed in MRSA252 cells treated with linoleic acid when it was added to a final concentration of 0.1 mM for 20 min during late-exponential growth (linoleic acid challenge) compared to unexposed control cells; 213 genes were up-regulated (Table 1) and 179 genes were down-regulated (Table 2). When transcription was analysed for cells grown in the presence of a lower concentration of linoleic acid (0.01 mM) from the time of inoculation (linoleic acid growth exposure) a correspondingly smaller subset of genes displayed differential transcription; 37 genes were up-regulated (Table 3) and 28 genes were down-regulated (Table 4). Oleic acid differs from linoleic acid in its degree of unsaturation, containing one less double bond in the chain. When cells were grown under the conditions of oleic acid growth exposure, 20 genes were up-regulated (Table 5) and 23 genes were down-regulated (Table 6).

**Table 1:** MRSA252 genes up-regulated following the addition of linoleic acid (0.1 mM) to exponentially growing cells (linoleic acid challenge).

Group Functions	MRSA252 ORF	MRSA252 Gene	MRSA252 Gene Product	Fold Change Up Regulated	P-value
Virulence Factors and Regulators	SAR0156	*capF*	capsular polysaccharide synthesis enzyme	2.23	4.22E-02
	SAR0163	*capM*	capsular polysaccharide synthesis enzyme	2.23	3.20E-02
	SAR0164	*capN*	capsular polysaccharide synthesis enzyme	2.83	1.79E-02
	SAR0625	*sarA*	staphylococcal accessory regulator A	2.14	1.50E-02
	SAR0842	*clfA*	clumping factor	4.12	6.58E-03
	SAR2122	*hld*	delta-hemolysin precursor	3.28	1.23E-02
	SAR2295		putative exported MAP/eap domain protein	3.21	8.77E-04
	SAR2443	*tcaR*	MarR family regulatory protein	3.15	1.76E-03
	RNAIII	RNAIII	RNAIII accessory gene regulator (*agr*) locus	2.01	3.02E-02
Stress Response	SAR0577	*proP*	putative proline/betaine transporter	8.31	5.78E-04
	SAR0859		putative organic hydroperoxide resistance protein	3.82	1.23E-02
	SAR0938	*clpB*	putative ATPase subunit of an ATP-dependent protease	2.49	8.15E-04
	SAR1344	*katA*	catalase	5.71	1.86E-03
	SAR1656	*dnaJ*	chaperone protein	2.30	4.25E-02
	SAR1657	*dnaK*	chaperone protein	2.41	2.17E-03
	SAR2273	*asp23*	alkaline shock protein 23	2.06	3.86E-02
	SAR2276	*opuD2*	glycine betaine transporter 2	4.42	6.16E-03
	SAR2561		alkylhydroperoxidase, AhpD family	6.83	8.77E-04
	SAR2628	*clpL*	putative ATPase subunit of an ATP-dependent protease	4.06	4.79E-03
Energy Metabolism	SAR0113	*lldP1*	L-lactate permease 1	2.15	1.07E-03
	SAR0188		putative isochorismatase	4.91	8.03E-04
	SAR0141	*drm*	putative phosphopentomutase	2.45	1.31E-02
	SAR0574		putative hexulose-6-phosphate synthase	2.36	1.80E-03
	SAR0575		putative 6-phospho-3-hexuloisomerase	2.16	5.11E-03
	SAR0775		Osmoprotectant ABC transporter	2.13	4.80E-03
	SAR0776		Osmoprotectant ABC transporter, permease protein	2.99	3.00E-04
	SAR0824		putative malolactic enzyme	2.59	9.27E-03
	SAR0830	*tpiA*	triosephosphate isomerase	2.22	3.39E-02
	SAR0831	*pgm*	putative phosphoglycerate mutase	2.64	1.39E-02
	SAR1017	*menD*	putative menaquinone biosynthesis bifunctional protein	2.24	1.65E-03
	SAR1018		putative hydrolase	2.80	1.65E-03
	SAR2386		putative NAD-dependent dehydrogenase	3.73	3.00E-04
	SAR2506	*dpgm*	putative phosphoglycerate mutase	2.06	7.33E-04
	SAR2684	*fda*	fructose-bisphosphate aldolase class I	2.02	5.85E-03
	SAR2687		putative AMP-binding enzyme	2.01	9.65E-03
	SAR2724		isochorismatase family protein	3.00	8.30E-04
DNA Repair and Replication	SAR0363	*ssb*	putative single-strand DNA-binding protein	2.26	3.29E-03
	SAR0744		putative DNA photolyase	3.46	6.97E-04
	SAR0813	*uvrA*	excinuclease ABC subunit A	2.45	2.06E-03
	SAR0836	*rnr*	putative ribonuclease R	3.40	3.55E-03
	SAR0837	*smpB*	putative tmRNA-binding protein	3.07	3.70E-04
Protein Synthesis	SAR0364	*rpsR*	30S ribosomal protein S18	2.40	1.99E-02
	SAR0552	*fus*	translation elongation factor G	2.10	3.39E-02
	SAR1638	*rpoD*	RNA polymerase sigma factor	2.86	3.70E-04
	SAR2308	*rplQ*	50S ribosomal protein L17	2.60	1.99E-02
	SAR2309	*rpoA*	DNA-directed RNA polymerase alpha chain	2.36	3.35E-02
	SAR2310	*rpsK*	30S ribosomal protein S11	2.46	3.77E-02
	SAR2311	*rpsM*	30S ribosomal protein S13	2.45	3.12E-02
	SAR2313	*infA*	translation initiation factor IF-1	2.08	1.49E-02
	SAR2728		preprotein translocase SecA subunit-like protein	3.85	6.58E-03
Peptidoglycan Synthesis	SAR0878	*csdB*	putative selenocysteine lyase	2.52	3.07E-02
	SAR1026	*atl*	bifunctional autolysin precursor	2.65	6.16E-03
	SAR1158	*mraY*	phospho-N-acetylmuramoyl-pentapeptide-transferase	2.13	9.56E-04
	SAR1159	*murD*	UDP-N-acetylmuramoylalanine–D-glutamate ligase	2.39	7.64E-03
	SAR1160		putative cell division protein	2.10	1.31E-02
	SAR1290		putative exported CHAP domain protein	3.17	6.97E-04
	SAR1430	*murG*	putative N-acetylglucosamine transferase	5.26	1.86E-03
	SAR1761	*lysP*	lysine-specific permease	2.07	3.01E-02
	SAR2109	*dapE*	putative succinyl-diaminopimelate desuccinylase	4.89	3.00E-03
	SAR2188	*murA1*	putative carboxyvinyltransferase	2.94	6.54E-03
	SAR2269		putative alanine racemase	2.64	1.78E-03
	SAR2346	*fmhB*	putative pentaglycine interpeptide biosynthesis protein	2.49	4.22E-03
	SAR2394		putative protein associated with cell-envelope regulation	2.34	2.55E-03
	SAR2420	*hutG*	arginase family protein	2.83	4.71E-03
	SAR2521		putative membrane GtrA-like protein	3.11	5.78E-04
Fatty Acid Metabolism	SAR1438		conserved hypothetical protein	2.64	4.94E-03
	SAR2187	*fabZ*	putative hydroxymyristoyl-(acyl carrier protein) dehydratase	2.41	4.22E-02
Carotenoid Biosynthesis	SAR0596	*mvaK1*	mevalonate kinase	2.32	3.00E-04
	SAR0597	*mvaD*	mevalonate diphosphate decarboxylase	3.35	9.23E-04
	SAR0598	*mvaK2*	phosphomevalonate kinase	3.18	5.09E-04
	SAR2642	*crtN*	squalene synthase	4.95	8.03E-04
	SAR2643	*crtM*	squalene desaturase	7.18	2.38E-02
	SAR2645	*crtQ*	putative glycosyl transferase	6.07	3.00E-03
	SAR2646	*crtP*	putative phytoene dehydrogenase related protein	6.28	1.73E-03
	SAR2647		putative membrane protein	4.47	1.73E-03
Antibiotic Resistance	SAR0139		putative tetracycline resistance protein	4.06	1.59E-03
	SAR1622		metallo-beta-lactamase superfamily protein	2.08	3.93E-03
	SAR1785		metallo-beta-lactamase superfamily protein	3.05	1.08E-03
	SAR1831	*blaZ*	beta-lactamase precursor	2.02	2.72E-02
	SAR2505	*mdeA*	putative transport system protein	3.93	7.74E-03
	SAR2558		conserved hypothetical beta-lactamase-like protein	8.72	3.70E-04
	SAR2632		Putative MMPL efflux pump	2.03	4.58E-02
	SAR2655		putative glyoxalase	5.15	1.11E-03
	SAR2668		hypothetical aminoglycoside phosphotransferase protein	4.35	6.30E-03
Miscellaneous	SAR1738	*tnpB2*	transposase B 2	2.14	1.25E-03
	SAR2725	*sasF*	putative surface anchored protein	16.80	4.68E-05
Metabolism	SAR0108		putative peptidase	2.98	5.22E-03
	SAR0109		putative transporter protein	2.37	1.52E-02
	SAR0170		putative cation efflux system protein	2.50	1.77E-03
	SAR0306		ABC transporter ATP-binding protein	6.10	1.68E-03
	SAR0324		putative lipoate-protein ligase A	2.09	4.31E-03
	SAR0325		putative reductase	4.80	8.17E-04
	SAR0556		ThiJ/PfpI family protein	7.20	7.59E-04
	SAR0589		putative amino acid permease	4.19	3.75E-03
	SAR0600		pyridine nucleotide-disulphide oxidoreductase protein	2.26	2.77E-04
	SAR0624		putative esterase	6.49	7.59E-04
	SAR0729		putative acetyltransferase	2.92	3.23E-03
	SAR0732		putative acetyltransferase	2.34	3.00E-04
	SAR0756		aldo/keto reductase family protein	2.96	6.16E-04
	SAR0757		putative glucosyl transferase	3.49	7.59E-04
	SAR0764		putative 6-pyruvoyl tetrahydropterin synthase	3.70	8.03E-04
	SAR0841		putative acetyltransferase	5.22	3.29E-03
	SAR0883		putative dioxygenase	5.40	1.75E-03
	SAR0903		putative pyridine nucleotide-disulphide oxidoreductase	2.82	3.00E-04
	SAR0953		transport system extracellular binding lipoprotein	2.18	6.05E-03
	SAR1076		Spermidine/putrescine-binding protein homolog.	4.46	8.77E-04
	SAR1247		putative tRNA pseudouridine synthase B	2.31	2.10E-03
	SAR1340	*thrB*	homoserine kinase	2.20	3.71E-02
	SAR1431		putative acetyltransferase	4.58	1.80E-03
	SAR1439	*dfrB*	dihydrofolate reductase type I	2.13	6.34E-03
	SAR1440	*thyA*	thymidylate synthase	5.32	1.58E-03
	SAR1585	*malR*	maltose operon transcriptional repressor	2.21	2.20E-02
	SAR1655		putative methyltransferase	2.25	6.08E-03
	SAR2210		aldehyde dehydrogenase family protein	5.48	1.51E-03
	SAR2352	*moaA*	putative molybdenum cofactor biosynthesis protein A	2.07	1.86E-03
	SAR2385		putative Na+/H+ antiporter	2.35	1.34E-02
	SAR2395		inositol monophosphatase family protein	2.90	7.59E-04
	SAR2413		putative short chain dehydrogenase	4.63	3.66E-03
	SAR2460		putative acetyltransferase (GNAT) family protein	2.26	6.97E-04
	SAR2485	*narH*	nitrate reductase beta chain	2.16	1.02E-02
	SAR2541		putative carboxylesterase	2.45	1.79E-02
	SAR2544		ABC transporter ATP-binding protein	6.01	1.68E-03
	SAR2559		putative short chain dehydrogenase	6.85	6.16E-04
	SAR2659		putative short chain dehydrogenase	2.65	1.76E-03
	SAR2661		putative hydrolase	8.11	8.27E-04
	SAR2754	*hisIE*	putative histidine biosynthesis bifunctional protein	2.09	2.19E-02
	SAR2778		putative nickel transport protein	2.51	3.59E-03
Hypothetical Genes	SAR0111		putative myosin-crossreactive antigen	5.96	2.43E-03
	SAR0112		putative membrane protein	3.57	4.80E-04
	SAR0171		hypothetical protein	2.64	3.01E-02
	SAR0269		LacI family regulatory protein	2.57	3.59E-03
	SAR0299		hypothetical protein	2.05	4.99E-02
	SAR0305		putative membrane protein	3.89	6.02E-03
	SAR0390		putative lipoprotein	3.97	1.68E-03
	SAR0392		putative membrane protein	2.54	1.20E-02
	SAR0405		hypothetical protein	2.76	1.07E-02
	SAR0444		putative lipoprotein	2.31	2.16E-03
	SAR0498	*yabJ*	putative regulatory protein	3.65	1.07E-03
	SAR0499	*spoVG*	stage V sporulation protein G	2.83	1.11E-02
	SAR0601		putative DNA-binding protein	2.15	4.48E-04
	SAR0670		putative sensor histidine kinase protein	2.01	4.80E-04
	SAR0721		multicopper oxidase protein	2.29	4.39E-03
	SAR0733		conserved hypothetical protein	3.04	1.99E-03
	SAR0734		conserved hypothetical protein	2.23	1.59E-03
	SAR0821		conserved hypothetical protein	3.19	6.54E-03
	SAR0825		conserved hypothetical protein	5.06	1.86E-03
	SAR0840		putative membrane protein	5.25	2.63E-03
	SAR0849		hypothetical protein	2.81	6.05E-03
	SAR0850		hypothetical protein	2.94	6.81E-04
	SAR0854		hypothetical protein	4.07	1.56E-02
	SAR0855		hypothetical protein	2.53	1.94E-03
	SAR0867		hypothetical protein	2.54	4.55E-03
	SAR0877		conserved hypothetical protein	2.34	3.01E-02
	SAR0879		NifU-like protein	2.06	3.39E-02
	SAR0880		conserved hypothetical protein	2.14	3.47E-03
	SAR0882		putative membrane protein	4.05	4.12E-03
	SAR0931		putative membrane protein	7.87	4.28E-04
	SAR1055		hypothetical protein	4.50	3.08E-03
	SAR1077		putative membrane protein	2.54	5.69E-03
	SAR1227		conserved hypothetical protein	2.11	1.71E-02
	SAR1258		putative DNA-binding protein	2.12	3.07E-04
	SAR1289		putative exported protein	3.49	1.65E-03
	SAR1306		hypothetical protein	2.20	5.66E-03
	SAR1429		putative membrane protein	5.74	1.84E-03
	SAR1528		hypothetical phage protein	6.04	4.99E-02
	SAR1623		conserved hypothetical protein	2.29	1.39E-02
	SAR1669		conserved hypothetical protein	2.01	1.03E-02
	SAR1670		conserved hypothetical protein	2.53	5.36E-03
	SAR1671		probable nicotinate-nucleotide adenylyltransferase	2.03	8.07E-03
	SAR1816		putative membrane protein	2.82	6.16E-04
	SAR1854		hypothetical protein	4.98	1.27E-03
	SAR1965		ThiJ/PfpI family protein	2.25	4.83E-02
	SAR1970		conserved hypothetical protein	2.17	4.68E-02
	SAR1972		putative exported protein	5.71	6.59E-03
	SAR2010		hypothetical protein	3.49	6.58E-03
	SAR2047		hypothetical phage protein	2.12	1.16E-02
	SAR2085		hypothetical phage RecT family protein	2.18	9.84E-04
	SAR2088		hypothetical phage protein	2.62	2.28E-02
	SAR2094		hypothetical phage protein	2.69	3.06E-03
	SAR2095		hypothetical phage protein	4.03	5.44E-03
	SAR2098		hypothetical phage protein	2.02	2.87E-03
	SAR2189		putative membrane protein	2.94	6.07E-03
	SAR2232		conserved hypothetical protein	8.26	2.06E-03
	SAR2245		putative transcriptional antiterminator	6.03	1.76E-03
	SAR2270		hypothetical IucA/IucC family protein	3.36	3.80E-03
	SAR2274		putative membrane protein	4.59	2.04E-03
	SAR2275		putative membrane protein	3.98	6.16E-04
	SAR2347		putative membrane protein	2.21	6.54E-03
	SAR2392		conserved hypothetical protein	3.03	5.20E-03
	SAR2393		putative molydopterin dinucleotide binding domain protein	3.26	2.50E-03
	SAR2444		putative membrane protein	4.38	2.77E-04
	SAR2469		putative pyridoxamine 5′-phosphate oxidase	4.72	1.11E-03
	SAR2496		putative solute binding lipoprotein	2.60	3.66E-03
	SAR2525		hypothetical protein	5.28	2.33E-05
	SAR2532		CapD domain protein	2.48	4.16E-03
	SAR2542		putative transport protein	2.01	5.64E-03
	SAR2543		putative membrane protein	6.37	6.16E-04
	SAR2568		hypothetical protein	4.66	1.65E-03
	SAR2656		conserved hypothetical protein	3.35	6.16E-04
	SAR2657		hypothetical protein	2.40	8.80E-04
	SAR2658		TetR family regulatory protein	2.22	4.39E-04
	SAR2660		conserved hypothetical protein	7.26	6.16E-04
	SAR2665		conserved hypothetical protein	2.19	4.06E-03
	SAR2666		hypothetical protein	2.75	1.87E-03
	SAR2667		hypothetical protein	2.19	1.37E-02
	SAR2688		hypothetical protein	7.55	3.70E-04
	SAR2689		hypothetical protein	2.53	1.84E-02
	SAR2726		conserved hypothetical protein	5.07	1.80E-03
	SAR2727		glycosyl transferase, group 1 family protein	4.11	6.70E-03
	SAR2739		conserved hypothetical protein	4.21	2.06E-03
	SAR2740		conserved hypothetical protein	2.05	3.09E-02
	SAR2777		putative DNA-binding protein	2.40	1.90E-03
	SAR2780		putative membrane protein	7.38	4.80E-04

**Table 2:** MRSA252 genes down-regulated following the addition of linoleic acid (0.1 mM) to exponentially growing cells (linoleic acid challenge).

Group Functions	MRSA 252 ORF	MRSA252 Gene	MRSA252 Gene Product	Fold Change Down Regulated	P-value
Virulence Factors and Regulators	SAR0105	*plc*	1-phosphatidylinositol phosphodiesterase	3.85	4.39E-04
	SAR1574	*fur*	iron uptake regulatory protein	2.08	2.46E-02
	SAR1984		ferritin	2.56	4.39E-03
	SAR2001		staphopain protease	2.44	3.00E-04
	SAR2474	*sarZ*	MarR family virulence regulator	2.22	2.18E-02
	SAR2155	*rsbU*	putative sigma factor sigB regulation protein	2.56	1.58E-03
	SAR2715	*argR*	arginine repressor family protein	2.27	4.27E-03
	SAR2716	*aur*	zinc metalloproteinase aureolysin precursor	2.17	1.76E-03
Energy Metabolism	SAR0234	*ldh1*	L-lactate dehydrogenase 1	2.94	2.99E-05
	SAR0235		putative PTS system, IIBC component	2.22	1.30E-03
	SAR0242		putative galactitol PTS component	2.13	1.76E-02
	SAR0263		putative PTS system protein	2.50	3.44E-02
	SAR0355		Cys/Met metabolism PLP-dependent enzyme	2.04	2.74E-02
	SAR0522		putative pyridoxal 5-phosphate biosynthesis protein	3.03	3.00E-04
	SAR0523		SNO glutamine amidotransferase family protein	2.70	3.70E-04
	SAR0752		putative phosphofructokinase	2.38	3.27E-02
	SAR0753	*fruA*	fructose-specific PTS system component	2.50	3.78E-02
	SAR0766		glutamine amidotransferase class-I protein	2.04	6.16E-04
	SAR1088		putative pyruvate carboxylase	2.5	8.78E-04
	SAR1450	*tdcB*	putative threonine dehydratase	2.22	5.47E-03
	SAR1451	*ald2*	alanine dehydrogenase 2	3.03	1.15E-03
	SAR1777	*pfkA*	6-phosphofructokinase	2.86	2.87E-03
	SAR1789	*ackA*	acetate kinase	2.33	9.23E-03
	SAR2143	*ilvC*	ketol-acid reductoisomerase	2.22	2.80E-02
	SAR2213	*fba*	putative tagatose-bisphosphate aldolase	3.13	6.59E-03
	SAR2262		putative uridylyltransferase	2.27	8.35E-03
	SAR2579	*gtaB*	putative uridylyltransferase	2.63	6.84E-03
	SAR2720		putative PTS system component	4.17	3.54E-03
	SAR2721	*pmi*	mannose-6-phosphate isomerase	3.57	2.76E-03
Cell Wall Synthesis	SAR0228		putative glutamine amidotransferase class-I	2.13	2.08E-03
	SAR0257	*lytS*	autolysin sensor kinase protein	3.33	3.00E-03
	SAR0258	*lytR*	autolysin response regulator protein	3.57	9.15E-03
	SAR0259	*lrgA*	holin-like protein	2.22	1.20E-03
	SAR0646	*tagA*	teichoic acid biosynthesis protein	2.78	6.80E-03
	SAR0648	*tagG*	teichoic acid ABC transporter permease protein	2.38	1.32E-02
	SAR0649	*tagB*	teichoic acid biosynthesis protein	2.50	4.41E-04
	SAR1143		putative carbamate kinase	2.27	6.79E-03
	SAR1752	*hemA*	glutamyl-tRNA reductase	2.27	3.93E-02
	SAR1807		putative transglycosylase	2.04	8.78E-04
	SAR2472	*gltT*	putative proton/sodium-glutamate symport protein	2.04	2.97E-02
	SAR2621	*cidA*	holin-like protein	2.27	1.18E-02
	SAR2669		putative dihydroorotate dehydrogenase	2.86	3.75E-03
Fatty Acid Metabolism	SAR0225	*fadD*	putative acyl-CoA dehydrogenase	2.17	3.27E-02
	SAR0227	*fadX*	putative acetyl-CoA transferase	2.13	4.95E-02
	SAR0803		conserved hypothetical protein	3.23	1.02E-02
Carotenoid Biosynthesis	SAR1278	*miaA*	putative isopentenylpyrophosphate transferase	2.00	9.23E-03
	SAR1479		putative heptaprenyl diphosphate synthase	2.78	1.62E-02
	SAR1480	*menH*	heptaprenylnaphthoquinone methyltransferase	2.27	3.54E-02
	SAR1481		putative hexaprenyl diphosphate synthase	3.13	1.79E-02
DNA Repair and Replication	SAR0001	*dnaA*	chromosomal replication initiator protein DnaA	2.04	2.17E-03
	SAR0004	*recF*	DNA replication and repair protein RecF	2.08	8.51E-03
	SAR0028	*repB*	replication protein (pseudogene)	4.35	1.48E-02
	SAR0485	*holB*	putative DNA polymerase III, delta' subunit	3.03	1.26E-02
	SAR0711		putative replication initiation protein	2.50	3.43E-02
	SAR2429		putative 3-methylpurine glycosylase	2.22	1.87E-03
Metabolism	SAR0246	*ispD*	conserved hypothetical protein	2.00	2.27E-03
	SAR0261		putative nitric oxide reductase	2.22	6.16E-04
	SAR0302		putative formate/nitrite transporter	2.38	8.03E-03
	SAR0524	*nupC*	nucleoside permease	2.94	3.96E-03
	SAR0562		putative deoxyadenosine kinase protein	2.17	2.64E-02
	SAR0563		putative deaminase	2.50	3.75E-03
	SAR0569		putative glycosyl transferase	2.13	4.40E-03
	SAR0642		ABC transporter permease protein	2.56	9.65E-03
	SAR0643		ABC transporter ATP-binding protein	3.70	9.25E-03
	SAR0655		putative Na+ dependent nucleoside transporter	2.17	2.25E-03
	SAR0743		putative sodium:sulfate symporter protein	2.22	4.39E-04
	SAR0847	*nuc*	thermonuclease precursor	3.33	3.70E-04
	SAR0916		putative peptidyl-prolyl cis-trans isomerase	2.13	6.54E-03
	SAR1008		putative glycosyl transferases	4.00	1.65E-03
	SAR1014		acetyltransferase (GNAT) family protein	2.27	6.54E-03
	SAR1090	*ctaB*	putative protoheme IX farnesyltransferase	2.04	2.66E-02
	SAR1185		putative guanylate kinase	2.78	6.16E-03
	SAR1449		amino acid permease	2.50	2.65E-03
	SAR1478	*ndk*	putative nucleoside diphosphate kinase	2.38	3.03E-02
	SAR1598		arginine repressor	2.50	3.23E-03
	SAR1627		5-formyltetrahydrofolate cyclo-ligase family protein	2.78	2.27E-03
	SAR1707		putative ATPase	2.13	1.36E-02
	SAR1714	*relA*	GTP pyrophosphokinase	2.27	3.96E-03
	SAR1717	*secF*	putative protein-export membrane protein	2.27	6.05E-03
	SAR1804		putative acyltransferase	2.44	2.99E-02
	SAR2129	*scrR*	sucrose operon repressor	2.56	1.38E-02
	SAR2130		ammonium transporter family protein	2.04	1.65E-03
	SAR2340		acetyltransferase (GNAT) family protein	3.03	8.77E-04
	SAR2363	*modA*	putative molybdate-binding lipoprotein precursor	2.08	2.97E-02
	SAR2432		CorA-like Mg2+ transporter protein	2.44	5.61E-03
	SAR2493		putative formate/nitrite transporter	2.22	8.71E-03
	SAR2594		ABC transporter ATP-binding protein	2.38	1.65E-03
	SAR2789		putative subtilase family protease	2.04	2.27E-03
Hypothetical Genes	SAR0013		putative membrane protein	2.17	1.81E-02
	SAR0024		conserved hypothetical protein	3.03	2.27E-03
	SAR0030		hypothetical protein	2.38	6.16E-03
	SAR0048		putative membrane protein	2.08	1.08E-02
	SAR0061		putative membrane protein	2.08	4.25E-02
	SAR0063		hypothetical protein	2.56	1.02E-02
	SAR0075		hypothetical protein	2.04	6.16E-04
	SAR0078		hypothetical protein	2.08	9.65E-03
	SAR0097		putative DNA-binding protein	2.17	2.99E-03
	SAR0145		putative lipoprotein	2.13	1.56E-02
	SAR0197		hypothetical protein	286	2.14E-02
	SAR0216		putative lipoprotein	2.04	6.16E-04
	SAR0338		putative membrane protein	2.86	2.40E-03
	SAR0383		abortive infection bacteriophage resistance related	4.76	1.99E-02
	SAR0618		putative iron compound-binding protein	2.27	4.08E-02
	SAR0673		conserved hypothetical protein	2.70	4.55E-03
	SAR0694		putative bacteriocin	2.38	3.75E-03
	SAR0695		putative bacteriocin-immunity membrane protein	2.22	2.08E-03
	SAR0718		putative membrane protein	3.33	9.93E-04
	SAR0761		putative lipoprotein	2.86	3.00E-04
	SAR0793		hypothetical protein	2.56	1.58E-02
	SAR0846		secreted von Willebrand factor-binding homolog	2.17	1.94E-02
	SAR0890		conserved hypothetical protein	2.56	8.06E-04
	SAR0893		putative membrane protein	2.13	4.74E-02
	SAR0898		conserved hypothetical protein	2.70	1.59E-02
	SAR0899		conserved hypothetical protein	2.33	4.94E-03
	SAR0915		kinase-associated protein B	2.44	8.06E-04
	SAR0970		protozoan/cyanobacterial globin family protein	2.38	1.11E-02
	SAR0971		conserved hypothetical protein	2.78	1.87E-03
	SAR0979		putative membrane protein	2.50	2.25E-03
	SAR0981		putative esterase	2.44	1.55E-03
	SAR0982		putative restriction-modification system protein	2.44	1.37E-03
	SAR0983		putative restriction-modification system protein	2.56	2.42E-03
	SAR0985		putative 2′,5′ RNA ligase family	2.13	2.32E-02
	SAR0987		putative monogalactosyldiacylglycerol synthase	2.56	6.54E-03
	SAR1066		putative lipoprotein	2.50	4.74E-02
	SAR1085		conserved hypothetical protein	2.33	3.44E-02
	SAR1086		conserved hypothetical protein	3.45	6.54E-03
	SAR1095		conserved hypothetical protein	2.86	2.16E-02
	SAR1114		putative cell division protein ZapA	2.38	3.96E-03
	SAR1148		putative DNA-binding protein	2.38	2.66E-02
	SAR1154		MraZ protein	2.50	3.00E-03
	SAR1312		hypothetical protein	3.85	3.27E-02
	SAR1315		hypothetical protein	2.38	2.99E-03
	SAR1316		hypothetical protein	2.27	1.79E-02
	SAR1320		hypothetical protein	4.00	1.46E-02
	SAR1335		putative exported protein	2.27	7.38E-03
	SAR1389		conserved hypothetical protein (pseudogene)	2.33	5.63E-03
	SAR1448		major facilitator superfamily transporter protein	2.04	4.57E-03
	SAR1556		putative phage regulatory protein	2.08	5.64E-03
	SAR1558		putative phage lipoprotein	2.44	1.24E-03
	SAR1559		hypothetical phage protein	2.33	4.80E-04
	SAR1560		hypothetical phage protein	2.04	1.72E-02
	SAR1561		putative phage membrane protein	2.13	1.10E-03
	SAR1581		conserved hypothetical protein	2.86	1.81E-02
	SAR1592		conserved hypothetical protein	2.27	1.16E-02
	SAR1699		conserved hypothetical protein	2.00	3.92E-03
	SAR1706		putative transcriptional regulator	3.45	2.16E-02
	SAR1708		conserved hypothetical protein	2.04	3.14E-03
	SAR1770		putative membrane protein	2.13	2.99E-03
	SAR1834		putative leucyl-tRNA synthetase	2.17	3.65E-02
	SAR1885		hypothetical protein	2.63	6.97E-04
	SAR1897		hypothetical protein	3.03	2.86E-02
	SAR1935		probable phosphoesterase	2.78	2.37E-03
	SAR1938		putative DNA-binding protein	2.38	2.80E-02
	SAR2020		putative membrane protein	2.44	6.39E-03
	SAR2035		putative exported protein	2.86	1.61E-02
	SAR2113		hypothetical protein	2.86	4.64E-02
	SAR2114		hypothetical protein	2.56	3.43E-02
	SAR2115		hypothetical protein	2.86	4.22E-02
	SAR2118		putative membrane protein	2.00	3.28E-03
	SAR2119		membrane anchored protein	2.44	1.08E-03
	SAR2156		pemK-like protein	3.03	5.61E-03
	SAR2219		hypothetical protein	2.78	6.21E-03
	SAR2261		putative membrane protein	2.08	6.02E-04
	SAR2263		putative membrane protein	2.17	2.89E-03
	SAR2299		hypothetical protein	2.04	3.47E-03
	SAR2369		putative acyl-CoA dehydrogenase	2.86	1.94E-03
	SAR2425		putative membrane protein	2.13	5.63E-03
	SAR2428		putative membrane protein	2.00	3.23E-03
	SAR2435		putative acyl hydrolase	2.50	3.02E-02
	SAR2439		tetR family regulatory protein	2.22	3.23E-03
	SAR2473		putative exported protein	3.85	3.97E-03
	SAR2500		putative lipoprotein	2.86	8.48E-04
	SAR2546		putative lipoprotein	3.13	2.76E-02
	SAR2595		putative membrane protein	2.78	1.65E-03
	SAR2718		putative exported protein	2.04	4.80E-04
	SAR2719		transcriptional regulator	3.13	3.07E-04
	SAR2792		putative membrane protein	3.85	2.78E-03
	SAR2793		putative membrane protein	3.70	8.17E-04

**Table 3:** MRSA252 genes up-regulated during growth in the presence of linoleic acid (0.01mM) (linoleic acid growth exposure).

Group Functions	MRSA252 ORF	MRSA252 Gene	MRSA252 Gene Product	Fold Change Up Regulated	P-value
Virulence Factors and Regulators	SAR0279	*esxA*	virulence factor esxA	5.93	7.03E-08
	SAR0280	*esaA*	putative membrane protein	4.32	4.53E-06
	SAR0281	*essA*	putative membrane protein	2.70	1.66E-03
	SAR0282	*esaB*	conserved hypothetical protein	2.65	3.35E-03
	SAR0284	*essC*	putative membrane protein	2.56	1.25E-03
	SAR0284v	*essC*	putative membrane protein	2.49	4.23E-03
	SAR2123	*agrB*	putative autoinducer processing protein	9.36	1.74E-05
	SAR2125	*agrC*	autoinducer sensor protein	5.39	4.33E-05
	SAR2126	*agrA*	autoinducer sensor protein response regulator protein	2.25	1.41E-03
	agrIII	*agrIII*	Class III accessory gene regulator (*agr*) locus	8.71	4.16E-06
	RNAIII	RNAIII	RNAIII accessory gene regulator (*agr*) locus	10.20	1.21E-05
Metabolism	SAR0150	*adhE*	putative aldehyde-alcohol dehydrogenase	2.25	1.67E-02
	SAR0190	*glcA*	glucose-specific PTS transporter protein, IIABC component	2.05	3.76E-02
	SAR0829	*pgk*	phosphoglycerate kinase	2.76	2.16E-03
	SAR0830	*tpiA*	triosephosphate isomerase	2.75	1.69E-03
	SAR0831	*pgm*	putative phosphoglycerate mutase	2.83	2.22E-03
	SAR0832	*eno*	putative enolase	2.15	5.88E-03
	SAR2296	*alsD*	putative acetolactate decarboxylase	2.43	3.32E-03
	SAR2297	*alsS*	putative acetolactate synthase	2.17	1.41E-03
	SAR2618	*glcB*	PTS system, glucose-specific IIABC component	2.78	1.41E-02
	SAR2711	*arcC*	carbamate kinase	2.40	3.41E-02
	SAR2712	*arcD*	arginine/ornithine antiporter	2.21	1.88E-02
	SAR2713	*arcB*	putative ornithine carbamoyltransferase	2.31	1.88E-02
	SAR2714	*arcA*	arginine deiminase	2.89	1.41E-02
Hypothetical Genes	SAR0111		putative myosin-crossreactive antigen	2.44	6.52E-05
	SAR0277		putative exported protein	3.76	4.22E-05
	SAR0278		putative CHAP domain protein	2.89	1.22E-04
	SAR0299		possible pseudogene	2.95	3.30E-03
	SAR0301		putative membrane protein	3.44	1.93E-03
	SAR0385		similar to putative pathogenicity island gene *orf3*	4.09	1.93E-03
	SAR0839		putative lipoprotein	3.36	5.41E-05
	SAR1564		hypothetical protein	2.09	5.32E-04
	SAR1565		putative lipoprotein	2.38	3.02E-03
	SAR2426		putative membrane protein	2.09	2.11E-03
	SAR2427		ABC transporter ATP-binding protein	2.14	4.82E-03
	SAR2428		putative membrane protein	3.73	1.21E-05
	SAR2569		hypothetical protein	6.01	4.75E-02

**Table 4:** MRSA252 genes down-regulated during growth in the presence of linoleic acid (0.01mM ) (linoleic acid growth exposure).

Group Functions	MRSA252 ORF	MRSA252 Gene	MRSA252 Gene Product	Fold Change Down Regulated	P-value
Stess Response	SAR0525	*ctsR*	stress regulatory protein	3.57	6.52E-05
	SAR0526		uvrB/uvrC domain protein	4.35	4.16E-06
	SAR0528	*clpC*	putative stress response-related Clp ATPase	4.17	5.47E-05
	SAR0823	*clpP*	putative ATP-dependent Clp protease proteolytic subunit	2.04	3.82E-04
	SAR0938	*clpB*	putative ATPase subunit of an ATP-dependent protease	9.09	5.72E-06
	SAR1657	*dnaK*	chaperone protein	2.94	5.41E-05
	SAR1658	*grpE*	GrpE protein	3.57	7.03E-08
	SAR2116	*groEL*	60 kDa chaperonin	2.44	1.05E-03
	SAR2117	*groES*	10 kDa chaperonin	2.78	1.92E-04
Metabolism	SAR0189		putative thiamine pyrophosphate enzyme	2.94	1.51E-04
	SAR0208		putative sugar transport system permease	2.94	2.68E-02
	SAR0209		putative oxidoreductase	4.75	1.21E-02
	SAR0210		putative oxidoreductase	9.09	3.75E-03
	SAR0527		putative phosphotransferase	4.55	7.03E-08
	SAR0752		putative phosphofructokinase	2.44	3.20E-02
	SAR0753	*fruA*	fructose-specific PTS system component	3.45	1.21E-02
	SAR1274	*glpF*	putative glycerol uptake facilitator protein	3.70	3.36E-03
	SAR1275	*glpK*	glycerol kinase	4.17	6.42E-04
	SAR1276	*glpD*	glycerol-3-phosphate dehydrogenase	7.69	4.83E-06
	SAR2244	*mtlA*	mannitol-specific PTS system component	2.08	4.75E-02
	SAR2445	*hrtA*	Heme-regulated transporter ATPase	2.94	3.11E-04
	SAR2594		ABC transporter ATP-binding protein	2.33	3.32E-03
Hypothetical Genes	SAR0100		putative membrane protein	2.56	2.28E-02
	SAR0211		conserved hypothetical protein	11.11	3.02E-03
	SAR0584	*vraX*	predicted role in ipenimen resistance	2.27	3.15E-02
	SAR0750		conserved hypothetical protein	2.22	1.32E-02
	SAR0939		LysR family regulatory protein	2.94	5.81E-05
	SAR2595		putative membrane protein	2.04	7.18E-03

**Table 5:** MRSA252 genes up-regulated during growth in the presence of oleic acid (0.01 mM) (oleic acid growth exposure).

Group Functions	MRSA252 ORF	MRSA252 Gene	MRSA252 Gene Product	Fold Change Up Regulated	P-value
Virulence Factors and Regulators	SAR0279	*esxA*	virulence factor EsxA	3.20	1.51E-05
	SAR0280	*esaA*	putative membrane protein	2.67	1.07E-04
	SAR2122	*hld*	delta-hemolysin precursor	6.02	5.53E-04
	SAR2123	*agrB*	putative autoinducer processing protein	6.54	3.55E-07
	SAR2125	*agrC*	autoinducer sensor protein	3.77	9.89E-05
	SAR2126	*agrA*	autoinducer sensor protein response regulator protein	2.01	2.21E-04
	agrIII	*agrIII*	Class III accessory gene regulator (*agr*) locus	6.30	1.12E-07
	RNAIII	*RNAIII*	RNAIII accessory gene regulator (*agr*) locus	7.02	3.21E-05
Metabolism	SAR0753	*fruA*	fructose-specific PTS system component	2.07	1.34E-02
	SAR2296	*alsD*	conserved hypothetical protein	2.05	4.08E-03
	SAR2297	*alsS*	putative acetolactate synthase	2.49	1.19E-03
	SAR2711	*arcC*	carbamate kinase	4.09	6.41E-03
	SAR2712	*arcD*	arginine/ornithine antiporter	3.55	7.34E-04
	SAR2713	*arcB*	putative ornithine carbamoyltransferase	3.41	1.97E-03
	SAR2714	*arcA*	arginine deiminase	4.03	3.28E-03
Hypothetical Genes	SAR0277		putative exported protein	2.00	7.31E-03
	SAR0301		putative membrane protein	2.15	2.54E-02
	SAR0385		putative membrane protein	2.88	8.02E-03
	SAR0839		putative lipoprotein	2.07	1.97E-03
	SAR1448		major facilitator superfamily	2.03	1.32E-02
	SAR2710		putative regulatory protein	2.62	6.93E-05

**Table 6:** MRSA252 genes down-regulated during growth in the presence of oleic acid (0.01 mM) (oleic acid growth exposure).

Group Functions	MRSA252 ORF	MRSA252 Gene	MRSA252 Gene Product	Fold Change Down Regulated	P-value
Stress Response	SAR0525	*ctsR*	stress regulatory protein	3.23	3.26E-05
	SAR0526		uvrB/uvrC domain protein	4.17	3.26E-05
	SAR0527		putative phosphotransferase	3.85	2.20E-07
	SAR0528	*clpC*	putative stress response-related Clp ATPase	3.23	1.01E-04
	SAR0938	*clpB*	putative ATPase subunit of an ATP-dependent protease	8.33	1.30E-06
	SAR1119	*uvrC*	putative excinuclease ABC subunit C	3.70	7.00E-03
	SAR1657	*dnaK*	chaperone protein	2.63	1.01E-04
	SAR1658	*grpE*	GrpE protein	2.86	4.78E-06
	SAR2116	*groEL*	60 kDa chaperonin	2.38	1.82E-03
	SAR2117	*groES*	10 kDa chaperonin	2.44	2.21E-04
Metabolism	SAR0120		putative ornithine cyclodeaminase	2.38	4.46E-02
	SAR0354		putative homocysteine S-methyltransferase	2.13	1.60E-02
	SAR0452		putative NADH-Ubiquinone protein	2.00	1.32E-02
	SAR1274	*glpF*	putative glycerol uptake facilitator protein	4.35	9.16E-03
	SAR1275	*glpK*	glycerol kinase	3.57	1.59E-02
	SAR1276	*glpD*	aerobic glycerol-3-phosphate dehydrogenase	4.76	3.26E-05
	SAR1849		proline dehydrogenase	3.23	8.02E-03
	SAR2445	*hrtA*	Heme-regulated transporter ATPase	2.94	1.01E-04
	SAR2446	*hrtB*	Heme-regulated transporter permease	2.22	3.98E-02
	SAR2582	*gntP*	putative gluconate permease	5.88	4.53E-03
	SAR2583	*gntK*	putative gluconokinase	4.55	3.60E-02
Hypothetical Genes	SAR0939		LysR family regulatory protein	2.86	2.76E-04
	SAR2581		hypothetical protein	4.55	3.99E-02

The sudden imposition of linoleic acid during exponential growth at OD_600_ = 3 (linoleic acid challenge) resulted in large-scale transcriptional reprogramming of genes in four major discernible categories, including: virulence, energy metabolism, stress resistance and cell wall synthesis. In contrast, the presence of linoleic at 0.01 mM, a non-growth limiting concentration (linoleic acid growth exposure), resulted in changes in transcription of fewer genes in the same categories, with the exception of cell wall synthesis.

### Effect of linoleic acid on *S. aureus* MRSA252 transcription

A distinctive feature of linoleic acid addition to cells of MRSA252 under both challenge and growth exposure conditions was observed to be the 10- and 2-fold up-regulation of the virulence regulator RNAIII, respectively (Table 1, 3). Previous studies have not reported changes in regulation of this locus after exposure to FFAs in *S. aureus* [34], [35]. Moreover, after linoleic acid challenge the virulence regulator *sarA* was up-regulated as was *clfA,* encoding clumping factor A and genes required for capsule formation (*capF, capM, capN*), while the genes encoding the proteases staphopain and aureolysin were down-regulated (Table 1, 2). Further virulence-associated loci up-regulated in the presence of linoleic acid during growth included the *esxA* locus encoding ESAT-6-like proteins and the genes coding for their synthesis/secretion [41] and *tcaR* that encodes a MarR-like regulator of SarS and SasF expression [42] (Table 3).

Many genes involved in sugar metabolism showed altered levels of regulation. In particular, several genes in the fructose and mannose metabolism pathways were down-regulated. These include *SAR0753* (*fruA*) and *SAR0752,* involved in the importation and phosphorylation of fructose, respectively. Genes with similar functions involving the importation and phosphorylation of glucose, mannose, maltose and galactitol, namely *SAR0235*, *SAR1777* (*pfkA*), *SAR2720*, *SAR2721* (*pmi*), *SAR0242* and *SAR0263* were also down-regulated. This could indicate an alteration of central metabolism via the action of the linoleic acid. Here, in concert with these changes, many genes in glycolysis were up-regulated, including *SAR2684* (*fda*), *SAR0830* (*tpiA*), *SAR0831* (*pgm*) and *SAR2506* (*dpgm*). In addition, the *SAR0141* (*drm*), *SAR0574* and *SAR0575* genes involved in pentose and glucuronate interconversions were up-regulated, which would increase the availability of substrates for glycolysis or pentose phosphate pathways. The down-regulation of the putative UTP-glucose-1-phosphate uridylyltransferases *SAR2262* and *SAR2579* (*gtaB*), which are predicted to catalyse the conversion of glucose-1-phosphate to UDP-glucose, would maintain the pool of phosphorylated glucose available for glycolysis.

In addition to increased transcription of genes encoding glycolytic enzymes, the cells exposed to a linoleic acid challenge alter metabolism to maintain levels of pyruvate. The up-regulation of *SAR0824* which encodes malate dehydrogenase (converting malate to pyruvate) is predicted to increase pyruvate levels. Concomitantly, there was down-regulation of genes involved in pyruvate utilisation, including *ldh1*, *SAR1088* (*pycA*), *ald2* and *SAR0355* converting pyruvate to lactate, oxaloacetate, alanine and cysteine, respectively. Reduced transcription of *SAR2143* (*ilvC*) could further lower the expenditure of cellular pyruvate via amino acid synthesis, and down-regulation of *SAR0522* and *SAR0523* encoding predicted enzymes utilising glyceraldehyde-3-phosphate would prevent diversion of this intermediate from glycolysis. The reduced importation of substrates for glycolysis would explain increased levels of glycolytic enzymes and modulation of other pathways to increase pyruvate production. Under such potentially energy starved conditions, the pool of pyruvate would be pushed toward energy creation at the expense of less critical pathways.

There was up-regulation of many genes involved in cellular stress responses, including the CtsR regulon genes *clpB*, *dnaJ* and *dnaK* suggesting that linoleic acid addition is perceived by *S. aureus* as a stressor. Moreover, the transcripts of several σ^B^-regulated genes were up-regulated, including *katA*, *asp23* and *clpL*, and the *crtM*, *crtN*, *crtO*, *crtP*, *crtQ* genes involved in staphyloxanthin biosynthesis. The mevalonate pathway generates the isopentenyl-diphosphate precursor for biosynthesis of this carotenoid, and the pathway genes *mvaK1*, *mvaD* and *mvaK2* were up-regulated accordingly (Table 1). Linoleic acid has been proposed to interfere with membrane function by increasing fluidity, which has the potential to perturb the electron transport chain. The production of carotenoids, which insert into the membrane has been reported to decrease fluidity and counteract the effect of LC-uFFAs [26]. In response to linoleic acid challenge the menaquinone biosynthesis pathway genes *SAR1017* (*menD*) and *SAR1018* involved in the conversion of chorismate to menaquinone (MK), and present in an operon with *menB,* were up-regulated indicating an increase in MK biosynthesis. This up-regulated MK synthesis could be a response to perturbation of the electron transport chain. The *SAR1479*, *SAR1480* (*menH*) and *SAR1481* genes synthesise heptaprenyl diphosphate for the isoprenoid moiety of MK-7, while *SAR1278* (*miaA*) is a predicted isopentenyl-pyrophosphate transferase. These genes were down-regulated, which is consistent with a reduction of the MK-7 isoprenolog. *S. aureus* synthesises various MK isoprenologs, up to MK-9, and alters their ratio in response to changes in temperature and oxygen levels [43].

Genes concerned with cell wall biosynthesis were observed to be modulated in linoleic acid challenge conditions but not in the growth exposure conditions. The genes *mraY*, *murD*, *murG* and *murA1* involved in the synthesis of the pentaglycine precursor in PG synthesis were up-regulated, as was *atl*, encoding the major cellular autolysin (Table 1) [44]. There was down-regulation of the two-component regulatory system *lytRS*, the holin-like *lrgA* and *cidA* and the putative transglycosylase *SAR1807,* which have cell wall modulatory roles (Table 2) [45], [46]. In addition to these changes, an assortment of transcriptionally modulated genes was observed, which would function to maintain the level of constituents for the PG-pentapeptide precursor. SAR2109 (*dapE*), which catalyses the formation of a substrate for lysine biosynthesis and the lysine-specific permease *SAR1761* (*lysP*) were up-regulated, and this would increase the pool of L-lysine in the cell. Up-regulation of *SAR2420 (hutG),* and down-regulation of *SAR2669* encoding a putative dihydroorotate dehydrogenase, *SAR0228* encoding a putative glutamine amidotransferase and *SAR1752* (*hemA*), in concert, would maintain glutamate levels within the cell. *SAR2269*, a putative alanine racemase, was up-regulated thereby increasing synthesis of D-alanine by isomerising L-alanine. The microarray data also revealed increased transcription of the *tagA*, *tagG* and *tagB* genes concerned with teichoic acid biosynthesis.

The fatty acid biosynthesis enzyme FabI was previously reported to be inhibited by linoleic acid and was therefore proposed to be a key target for its antibacterial activity [23]. Here, within fatty acid metabolism, only *fabZ* was up-regulated in linoleic acid challenge conditions, whereas *fadD*, *fadX* and *plc* were down-regulated. *fabZ* is directly downstream of *murA1* within a predicted operon which may explain why *fabZ* alone is up-regulated amongst the fatty acid biosynthesis genes.

### Quantitative Real-Time PCR

Confirmation of the microarray data was performed using qRT-PCR to test selected transcriptional changes of known genes from different functional subsets. To this end, the expression level of genes involved in staphyloxanthin synthesis (*crtM*), PG biosynthesis (*murG*, *cidA* and *lytR*), stress responses (*katA* and *clpB*), virulence (RNAIII, *sarA*, *arcA*, *hla* and *spa*) and fatty acid metabolism (*fabZ*, *fabI*, *fadD* and *fadA*) were analysed. In addition, the *sasF* gene was analysed to confirm the particularly high levels of transcript that were observed under the challenge experimental conditions. Most genes tested showed the same pattern of up- or down-regulation (Table 7) that was identified by microarray analysis under any given set of conditions. The only exceptions were the fatty acid degradation pathway genes *fadD* and *fadA*. While *fadD* was 2.15 fold down-regulated after linoleic acid challenge when analysed by microarray, this was identified as a 3.16 fold up-regulation when tested by qRT-PCR. The *fadA* gene lies within a predicted operon with fadD and would thus be co-regulated. A 3.1 fold up-regulation of *fadA* was similarly measured by qRT-PCR when the cells were challenged with linoleic acid, which supports the reproducibility of the qRT-PCR analysis of *fadD* and its likely operon arrangement with *fadA*. Therefore, with the exception of the *fad* operon, the microarray data was shown to be consistent when tested by qRT-PCR.

**Table 7:** qRT-PCR analysis of gene expression in MRSA252.

ORF	Gene	Linoleic Challenge	Linoleic Growth	Oleic Growth	Linoleic Growth	Oleic Growth
			OD_600_ = 3	OD_600_ = 3	OD_600_ = 8	OD_600_ = 8
SAR0114	*spa*	1.46 (0.37)	1.01 (0.07)	−1.08 (0.11)	−3.03 (0.14)	−2.92 (0.24)
SAR0223	*fadA*	3.10 (0.08)	nd	nd	nd	nd
SAR0225	*fadD*	3.16 (0.61)	−1.34 (0.03)	−1.88 (0.02)	−1.06 (0.03)	−1.01 (0.02)
SAR0258	*lytR*	−5.03 (0.01)	nd	nd	nd	nd
SAR0625	*sarA*	3.84 (0.81)	1.10 (0.11)	−1.12 (0.07)	1.52 (0.55)	2.05 (1.12)
SAR2621	*cidA*	−1.93 (0.02)	1.52 (0.03)	1.41 (0.03)	−2.75 (0.01)	−1.39 (0.02)
SAR0938	*clpB*	3.90 (0.08)	−8.55 (0.01)	−10.31 (0.01)	−3.16 (0.01)	−3.00 (0.01)
SAR0978	*fabI*	1.25 (0.02)	nd	nd	nd	nd
SAR1136	*hla*	−1.60 (0.20)	1.19 (0.19)	−1.71 (0.09)	6.38 (2.91)	7.88 (5.16)
SAR1344	*katA*	7.27 (0.18)	nd	nd	nd	nd
SAR1430	*murG*	7.59 (0.22)	nd	nd	nd	nd
SAR2187	*fabZ*	3.37 (0.64)	−1.10 (0.02)	1.40 (0.02)	1.10 (0.04)	1.20 (0.06)
SAR2643	*crtM*	3.72 (0.08)	nd	nd	nd	nd
SAR2714	*arcA*	1.61 (0.03)	1.86 (0.04)	2.17 (0.04)	−2.19 (0.02)	−1.50 (0.01)
SAR2725	*sasF*	31.86 (0.69)	nd	nd	nd	nd
	RNAIII	7.86 (0.15)	56.14 (1.40)	34.28 (0.74)	156.12 (6.95)	153.30 (2.81)

The values correspond to the fold change for each gene tested under the relevant fatty acid treatment conditions when compared to the untreated control. The standard deviation for each measurement is in parentheses. nd, not detemined. ORF indicates the gene locus in MRSA252 (http://www.genedb.org/genedb/saureusMRSA/).

The transcription of a subset of genes was examined by qRT-PCR during mid-exponential growth phase and late exponential-phase (OD_600_ = 8) (Table 7), to examine the potential effect of the increased levels of the density-signalling effector RNAIII on transcription of regulated genes (e.g. *spa*, *hla* and *sarA*). qRT-PCR analysis was performed on MRSA252 genes under linoleic and oleic acid growth exposure conditions. The RNAIII and *clpB* transcripts were consistently up- or down-regulated, respectively, at all of the points tested during growth; at OD_600_ = 8 RNAIII was massively up-regulated (>150-fold) in the presence of either linoleic or oleic acid. The transcription of *sarA* was up-regulated 1.5- to 2-fold in post-exponential phase in these conditions. Post-exponential transcription *of hla* was >6-fold higher after growth with either linoleic or oleic acid in comparison with the untreated control. Interestingly, this increase was moderate compared to that observed for RNAIII of the *agr* locus, which is known to up-regulate expression of *hla*. This reflects the complex regulation of *hla* and may be due to the increase in *sarA* levels.

Several genes showed fluctuations in relative transcript levels during the growth cycle. For example, *arcA* transcription varied over the different sample points, with gene up-regulation at OD_600_ = 3.0 for the linoleic growth experiment as per the microarray results. However, *arcA* was down-regulated in post-exponential phasef growth phase in the presence of linoleic acid.

The observation of increased expression of RNAIII, *hla* and *spa* in MRSA252 in response to LC-uFFAs is significantly different to previously published experiments for these transcripts in alternative strains [34], [35]. The expression of a large subset of genes, confirmed by qRT-PCR to be altered following exposure of MRSA252 to linoleic acid (Table 7), were subsequently examined in SH1000 to determine whether they were similarly regulated (Table 8). This revealed that in SH1000 the up- or down-regulation of several genes was in direct contrast to the pattern observed in MRSA252. For example, where both microarray data and qRT-PCR data showed that there was a large up-regulation of RNAIII after challenge or growth exposure in MRSA252, pronounced down-regulation was observed in SH1000 by qRT-PCR. Contrasts in regulation between MRSA252 and SH1000 were also observed for *sarA, spa* and *sasF*. However, several genes not predicted to be RNAIII-regulated, including *lytR*, *clpB*, *fabI*, *murG*, and *arcA* exhibited similar patterns of regulation in both strains under the conditions tested.

**Table 8:** qRT-PCR analysis of gene expression in SH1000.

ORF	Gene	Linoleic Challenge	Linoleic Growth	Oleic Growth
			OD_600_ = 3	OD_600_ = 3
SAR0114	*spa*	2.19 (0.09)	−1.94 (0.02)	1.66 (0.04)
SAR0258	*lytR*	−2.31 (0.02)	nd	nd
SAR0625	*sarA*	1.26 (0.05)	−3.79 (0.02)	−3.45 (0.03)
SAR0938	*clpB*	1.95 (0.08)	−2.11 (0.02)	−2.58 (0.03)
SAR0978	*fabI*	−1.20 (0.04)	nd	nd
SAR1136	*hla*	−3.60 (0.01)	−2.11 (0.02)	−2.58 (0.03)
SAR1430	*murG*	1.84 (0.11)	nd	nd
SAR2643	*crtM*	1.32 (0.05)	nd	nd
SAR2714	*arcA*	nd	2.19 (0.10)	4.39 (0.18)
SAR2725	*sasF*	1.49 (0.06)	nd	nd
	RNAIII	−1.79 (0.03)	−3.29 (0.01)	−1.95 (0.01)

The values correspond to the fold change for each gene tested under the relevant fatty acid treatment conditions when compared to the untreated control. The standard deviation for each measurement is in parentheses. nd, not detemined. ORF indicates the gene locus in MRSA252 (http://www.genedb.org/genedb/saureusMRSA/) that was tested in SH1000.

### Proteomic analysis

The proteome of MRSA252 was analysed by 2D-PAGE to identify protein expression changes in exponentially growing cells that were exposed to linoleic acid under the challenge conditions used for the microarray experiments. This analysis was performed to determine whether the large-scale transcriptional modulation described above was translated into a correspondingly large-scale proteomic shift. Under these conditions, 58 proteins were significantly (P≤0.05) up-regulated ≥2-fold and 15 proteins were significantly (P≤0.05) down-regulated ≥2-fold. MALDI-MS was used to identify the most intense protein spots on the gel corresponding to proteins that were modulated by linoleic acid, and the identities of 38 up-regulated and 5 down-regulated proteins were unambiguously determined (Table 9 and 10). There was strong agreement between the observed changes in protein expression due to linoleic acid challenge exposure and the encoded functions of the genes modulated in the microarray experiments. In terms of the assigned metabolic pathways, the interpreted effects of the fatty acid upon the cell were therefore corroborated. Proteins associated with stress responses and PG and MK biosynthesis were modulated in response to linoleic acid. Similarly, the CapA protein involved in capsule biosynthesis was up-regulated over 3-fold. From the proteomic data, challenge with linoleic acid resulted in up-regulation of glycolysis pathway proteins and those linked to pyruvate metabolism. Moreover, the proteomic data were often complementary to those from the microarrays. Several proteins within the glycolysis and pyruvate metabolism pathways were up-regulated (e.g Gap1, Pgi), whereas all glycolytic genes except *eno* were up-regulated in the microarray experiment. A few contradictions were observed between the microarray and proteomics data. The *ald2*, *ackA*, *ispD*, *SAR0985* and *SAR2369* proteins were determined by proteomics to be up-regulated but were down-regulated according to microarray analysis.

**Table 9:** MRSA252 proteins up-regulated following the addition of linoleic acid (0.1 mM) to exponentially growing cells (linoleic acid challenge).

Group Functions	MRSA252 ORF	MRSA252 Gene	MRSA252 Gene Product	Fold Change Up Regulated	P-value
Virulence Factors and Regulators	SAR2745	*capA*	Capsular polysaccharide biosynthesis protein	3.36	2.27E-04
Energy Metabolism	SAR0140	*deoC1*	deoxyribose-phosphate aldolase	4.34	2.49E-03
	SAR0217		formate acetyltransferase	2.30	1.50E-03
	SAR0394		phosphoglycerate mutase family protein	2.94	1.67E-02
	SAR0828	*gap1*	glyceraldehyde 3-phosphate dehydrogenase 1	2.07	3.53E-03
	SAR0924	*pgi*	glucose-6-phosphate isomerase	4.18	7.43E-03
	SAR1451	*ald2*	alanine dehydrogenase 2	2.13	1.32E-03
	SAR1789	*ackA*	acetate kinase	2.81	6.97E-04
	SAR2506	*dpgm*	putative phosphoglycerate mutase	2.15	3.52E-02
	SAR2685	*mqo2*	malate:quinone oxidoreductase	3.39	8.08E-03
DNA Repair and Replication	SAR1639	*dnaG*	DNA primase	2.66	2.61E-03
	SAR1996	*lig*	DNA Ligase	2.09	6.11E-04
Protein Synthesis	SAR0552	*fus*	elongation factor G	4.47	2.51E-04
	SAR0552	*fus*	elongation factor G	2.10	2.09E-02
	SAR1216	*trmD*	putative tRNA (guanine-7-)-methyltransferase	2.33	4.84E-04
	SAR1720	*queA*	S-adenosylmethionine:tRNA ribosyltransferase-isomerase	2.11	1.68E-02
	SAR2309	*rpoA*	RNA polymerase alpha subunit	2.61	2.40E-03
Peptidoglycan Synthesis	SAR0470	*lysR*	family regulatory protein	2.20	1.07E-02
	SAR1762	*thrS*	threonyl-tRNA synthetase	2.77	9.07E-04
	SAR1991	*gatB*	aspartyl/glutamyl-tRNA amidotransferase subunit B	2.25	3.52E-02
	SAR2201	*glyA*	serine hydroxymethyltransferase	3.55	8.43E-03
	SAR2201	*glyA*	serine hydroxymethyltransferase	2.15	3.74E-03
Carotenoid Biosynthesis	SAR1378		prephenate dehydrogenase	2.37	8.08E-03
Miscellaneous	SAR0218		putative pyruvate formate-lyase activating enzyme	2.60	3.46E-02
	SAR0403		putative DNA binding protein	2.72	3.48E-02
	SAR2007		putative oxygenase/mitric oxide synthase	2.04	7.13E-03
	SAR2007		putative nitric oxide synthase	2.66	2.38E-02
Metabolism	SAR0150	*adhE*	putative aldehyde-alcohol dehydrogenase	3.48	5.07E-03
	SAR0246	*ispD*	2-C-methyl-D-erythritol 4-phosphate cytidylyltransferase	2.43	4.84E-04
	SAR0246	*ispD*	2-C-methyl-D-erythritol 4-phosphate cytidylyltransferase	2.21	8.75E-03
	SAR0564		putative haloacid dehalogenase-like hydrolase	2.22	1.01E-02
	SAR1070	*pdhD*	dihydrolipoamide dehydrogenase	2.22	2.62E-02
	SAR2353	*mobA*	molybdopterin-guanine dinucleotide biosynthesis protein	3.12	8.32E-04
	SAR2513		6-carboxyhexanoate–CoA ligase	4.84	4.13E-05
	SAR2641		putative aminotransferase	2.07	1.54E-02
Hypothetical Proteins	SAR0985		putative RNA ligase protein	2.31	2.12E-02
	SAR2064		hypothetical phage protein	2.06	1.41E-03
	SAR2369		Acyl-CoA dehydrogenase-related protein	3.36	4.69E-03

**Table 10:** MRSA252 proteins down-regulated following the addition of linoleic acid (0.1 mM) to exponentially growing cells (linoleic acid challenge).

Group Functions	MRSA252 ORF	MRSA252 Gene	MRSA252 Gene Product	Fold Change Down Regulated	P-value
Protein Synthesis	SAR0927	*spsB*	signal peptidase Ib	5.88	9.88E-04
	SAR1755	*tig*	trigger factor	2.56	1.93E-02
	SAR2179		putative membrane protein	2.17	2.05E-02
Peptidoglycan Synthesis	SAR1284	*glnA*	glutamine synthetase	2.33	5.00E-02
Metabolism	SAR0814	*hprK*	kinase/phosphorylase	3.03	6.66E-03

In addition to linoleic acid, the effect of the skin-associated LC-uFFA hexadecenoic acid [C16:1 (n-6)] on the cellular proteome was studied to determine whether there was a common response to LC-uFFAs on *S. aureus* MRSA252. Analysis of 2D-SDS-PAGE gels revealed strong spot conservation for proteins exhibiting modulated expression in response to hexadecenoic acid compared to linoleic acid. Under challenge conditions with 0.1 mM hexadecenoic acid, 95 proteins were significantly (P≤0.05) up-regulated ≥2-fold and 7 proteins were significantly (P≤0.05) down-regulated ≥2-fold. MALDI-MS was used to identify 63 of the most intense protein spots on the gel corresponding to proteins that were modulated by linoleic acid and the identities of 56 up-regulated and 5 down-regulated proteins were unambiguously determined (Table 11 and 12). Many of the same proteins, or different proteins within the same metabolic pathways e.g. glycolysis and pyruvate metabolism, were identified after exposure to hexadecenoic acid and linoleic acid. This indicates that there is commonality in the metabolic response to the cellular perturbations caused by exposure to these LC-uFFAs, which differ in chain length, and the number and position of double bonds.

**Table 11:** MRSA252 proteins up-regulated following the addition of hexadecenoic acid (0.1 mM) to exponentially growing cells (hexadecenoic acid challenge).

Group Functions	MRSA252 ORF	MRSA252 Gene	MRSA252 Gene Product	Fold Change Up regulated	P-value
Stress Response	SAR2116	*groEL*	chaperonin	2.03	3.49E-02
	SAR2273	*asp23*	alkaline shock protein 23	2.89	8.90E-03
	SAR2461		pyridine nucleotide-disulphide oxidoreductase family protein	2.05	3.99E-02
	SAR2461		pyridine nucleotide-disulphide oxidoreductase family protein	2.00	2.13E-03
	SAR2461		pyridine nucleotide-disulphide oxidoreductase family protein	2.59	2.49E-02
Energy Metabolism	SAR0140	*deoC1*	deoxyribose-phosphate aldolase	4.21	4.86E-03
	SAR0394		phosphoglycerate mutase family protein	4.79	1.06E-03
	SAR0828	*gap1*	glyceraldehyde 3-phosphate dehydrogenase 1	2.86	2.51E-03
	SAR0830	*tpiA*	triosephosphate isomerase	3.40	4.42E-03
	SAR0832	*eno*	enolase	3.20	1.83E-02
	SAR0832	*eno*	enolase	3.09	4.60E-03
	SAR0832	*eno*	enolase	2.07	5.15E-03
	SAR0924	*pgi*	glucose-6-phosphate isomerase	2.31	2.73E-02
	SAR1068	*pdhB*	putative pyruvate dehydrogenase E1 component	2.72	2.29E-04
	SAR1068	*pdhB*	putative pyruvate dehydrogenase E1 component	2.72	2.29E-04
	SAR1121	*sdhA*	putative succinate dehydrogenase flavoprotein	2.24	1.36E-02
	SAR1451	*ald2*	alanine dehydrogenase 2	2.21	2.30E-03
	SAR2605	*ddh*	D-lactate dehydrogenase	3.11	8.87E-03
	SAR2685	*mqo2*	malate:quinone oxidoreductase	4.79	1.06E-03
	SAR2685	*mqo2*	malate:quinone oxidoreductase	3.13	2.18E-03
DNA Repair and Replication	SAR1997	*pcrA*	ATP-dependent DNA helicase	2.23	3.49E-02
Protein Synthesis	SAR0552	*fus*	translation elongation factor G	3.56	3.84E-02
	SAR0553	*tuf*	translation elongation factor Tu	2.65	3.41E-02
	SAR0553	*tuf*	elongation factor Tu	4.13	7.56E-03
	SAR1216	*trmD*	putative tRNA (guanine-7-)-methyltransferase	2.80	1.69E-03
	SAR1485	*rpsA*	putative 30S ribosomal protein S1	2.91	4.91E-02
	SAR1719	*tgt*	queuine tRNA-ribosyltransferase	2.01	4.96E-02
	SAR1720	*queA*	S-adenosylmethionine:tRNA ribosyltransferase-isomerase	2.49	1.44E-02
	SAR2309	*rpoA*	DNA-directed RNA polymerase subunit alpha	2.44	9.73E-03
Peptidoglycan Synthesis	SAR1048	*purD*	putative phosphoribosylamine–glycine ligase	3.98	2.08E-02
	SAR1762	*thrS*	threonyl-tRNA synthetase	2.51	1.51E-02
	SAR2212	*murA2*	UDP-N-acetylglucosamine 1-carboxyvinyltransferase	2.13	1.36E-02
Cell Division	SAR1795	*ezrA*	putative septation ring formation regulator	3.11	3.71E-03
	SAR1795	*ezrA*	putative septation ring formation regulator	2.56	5.16E-03
Miscellaneous	SAR0218		putative pyruvate formate-lyase activating enzyme	2.39	4.03E-03
	SAR0403		putative DNA-binding protein	2.84	8.32E-03
	SAR2007		putative oxygenase	2.85	2.92E-02
Metabolism	SAR0351	*thl*	acetyl-CoA acetyltransferase	2.85	2.59E-04
	SAR0351	*thl*	acetyl-CoA acetyltransferase	2.77	9.07E-04
	SAR0514		putative O-acetylserine (thiol)-lyase	2.41	4.43E-03
	SAR1142	*otc*	ornithine carbamoyltransferase	2.03	5.43E-03
	SAR2352	*moaA*	putative molybdenum cofactor biosynthesis protein A	3.73	6.34E-03
	SAR2352	*moaA*	putative molybdenum cofactor biosynthesis protein A	2.25	9.64E-03
	SAR2460		putative acetyltransferase (GNAT) family protein	5.37	6.22E-05
	SAR2460		putative acetyltransferase (GNAT) family protein	5.20	2.03E-02
	SAR2641		putative aminotransferase	2.12	8.47E-03
	SAR2694	*nrdG*	putative anaerobic ribonucleotide reductase activating protein	3.38	4.66E-04
	SAR2694	*nrdG*	putative anaerobic ribonucleotide reductase activating protein	2.48	3.43E-02
Hypothetical Proteins	SAR0246	*ispD*	2-C-methyl-D-erythritol 4-phosphate cytidylyltransferase	3.37	2.20E-04
	SAR0246	*ispD*	2-C-methyl-D-erythritol 4-phosphate cytidylyltransferase	2.90	1.69E-03
	SAR0985		putative RNA ligase protein	2.11	1.68E-02
	SAR1105	*isdD*	hypothetical protein	2.25	3.60E-03
	SAR2063		hypothetical phage protein	2.06	1.41E-03
	SAR2369		putative acyl-CoA dehydrogenase	2.61	2.43E-04
	SAR2545		M42 glutamyl aminopeptidase	2.08	1.72E-02
	SAR2674		hypothetical protein	2.45	5.46E-04

**Table 12:** MRSA252 proteins down-regulated following the addition of hexadecenoic acid (0.1 mM) to exponentially growing cells (hexadecenoic acid challenge).

Group Functions	MRSA252 ORF	MRSA252 Gene	MRSA252 Gene Product	Fold Change Down Regulated	P-value
Protein Synthesis	SAR0927	*spsB*	signal peptidase Ib	4.55	8.44E-03
Peptidoglycan Synthesis	SAR0920		NAD-specific glutamate dehydrogenase	2.17	8.21E-03
Miscellaneous	SAR2622	*lysR*	family regulatory protein	2.13	1.21E-02
Metabolism	SAR0483	*tmk*	putative thymidylate kinase	2.27	1.04E-03
	SAR1399	*pstB*	ABC transporter ATP-binding protein	3.23	1.80E-03

### Identification of survival mutants

**Figure 2:**
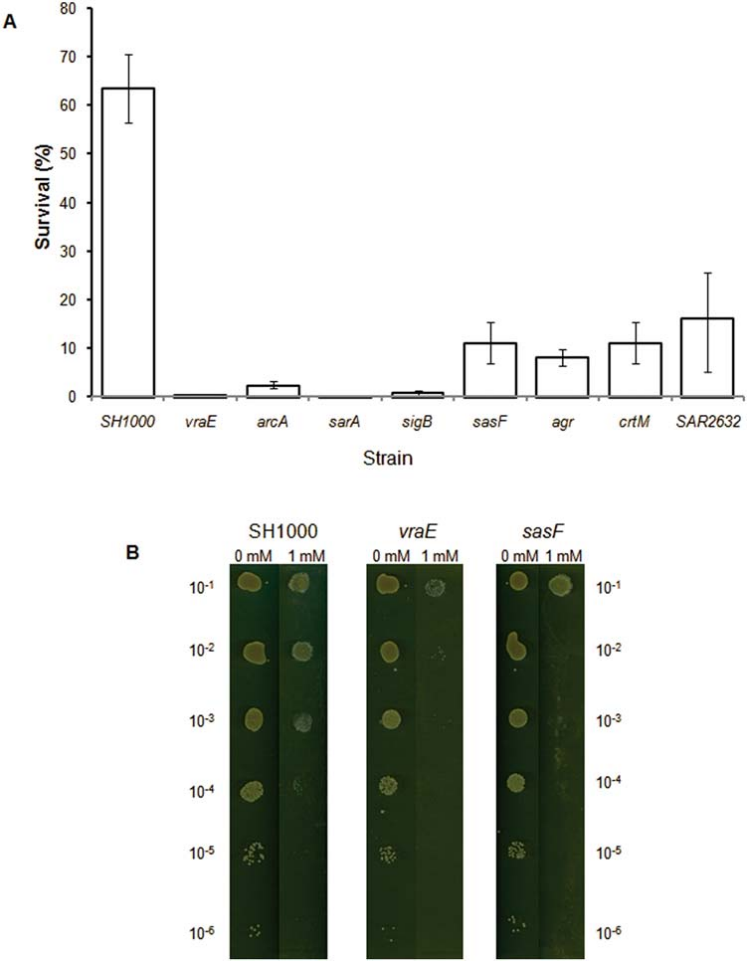
Plate based survival assay. A Graph showing the percentage survival of WT and mutant variants of SH1000 when serial dilutions of the strains were plated on BHI agar containing 1 mM linoleic acid. Survival is expressed as a percentage of viable cell counts obtained for control plates lacking linoleic acid. Values are the mean of multiple independent experiments. Error bars indicate standard errors of the mean. p<0.005 for each mutant by Student's t-test. B Plates showing the relative survival of SH1000 and the *sasF* (Liv694) and *vraE* (Liv753) mutants on BHI agar containing 0 or 1 mM linoleic acid. The 10^−1^ to 10^−6^ dilution series of cultures are as indicated.

Allelic replacement mutants were constructed in the genes *sasF* and *arcA*, which displayed altered transcription in response to linoleic acid and a further mutant was constructed in *vraS*, encoding a cell wall synthesis regulator. The contribution of these genes to survival in the presence of LC-uFFAs was tested on agar plates containing linoleic acid. Additionally, existing mutants of genes identified by microarray analysis to display altered transcription in response to linoleic acid, or regulators of these genes, were tested. Furthermore, a 5,000 clone Tn*917* mutant library was also screened to identify survival mutants. Analysis of the mutant clones was performed in SH1000 since MRSA252 is resistant to most antibiotics used for gene inactivation studies. Importantly, many of the mutant strains tested exhibited increased sensitivity to LC-uFFAs when compared to the wild-type, including those harbouring mutations in the genes: *sasF* (Liv694), *crtM* (Liv681), *arcA* (Liv692), *sigB* (Liv130), *agr* (Liv038) and *sarA* (Liv039) (Fig. 2A, 2B). In contrast *clfA* (Liv442), *vraS* (Liv718), *katA* (Liv750), *lytSR* (Liv101) and *clpC* (Liv671) did not contribute to survival under the conditions tested in a SH1000 background. Screening of the Tn*917* transposon library identified two further clones with defective survival. Sequencing upstream and downstream of the transposon in these mutants revealed insertion of Tn*917* in the *SAR2632* (Liv766) and *vraE* (Liv753) genes. Complementation of the fatty acid sensitivity of the *sasF*, *arcA*, *vraE* and *SAR2632* mutants was achieved by individually cloning each gene into the low copy number shuttle vector pSK5630 [75] and transforming each mutant with the relevant plasmid. Complementation restored survival of each mutant in LC-uFFA resistance assays (data not shown).

### Autolysis assays

**Figure 3:**
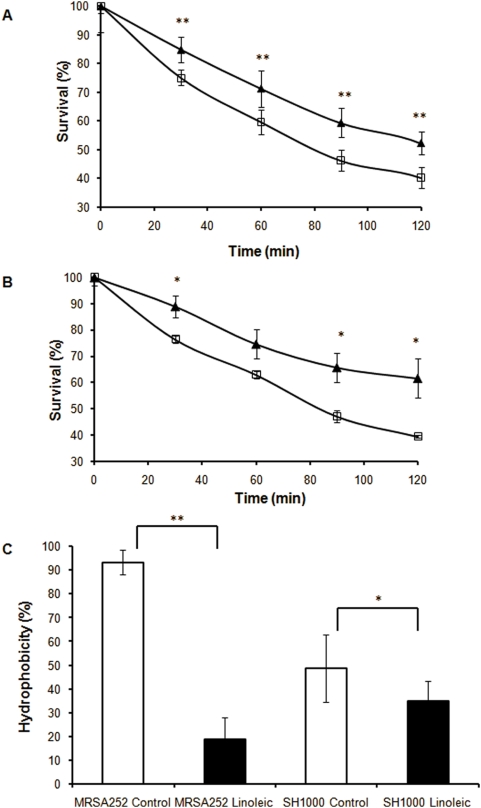
Physiological effects of linoleic acid on *S. aureus*. The result of growth of MRSA252 and SH1000 in the absence (closed triangle) or presence (open box) of 0.01mM linoleic acid on autolysis is shown in A and B, respectively. Survival is expressed as a percentage of OD_600_ at T = 0. Values are from three independent experiments. Error bars indicate standard errors of the mean. **p<0.01, *p<0.05 by Student's t test. C Relative hydrophobicity of the MRSA252 and SH1000 strains following overnight growth in BHI +/− 0.1 mM linoleic acid. Values are from three independent experiments. Error bars indicate standard errors of the mean. **p<0.01, *p<0.05 by Student's t test.

Cells grown in the presence of linoleic acid under constant growth conditions displayed reduced expression of the CtsR regulon, which is known to impact on cell autolysis [47]. Consequently we addressed the impact of the presence of LC-uFFAs upon autolysis of treated and control cells of MRSA252 and SH1000. A significantly increased rate of autolysis was observed in linoleic acid treated cells of each strain (Fig. 3A, 3B). This increase is in accordance with the reduced expression of the CtsR regulon in treated cells.

### Cell hydrophobicity

IsdA reduces cell surface hydrophobicity and acts to increase staphylococcal resistance to LC-uFFAs [34] while a GML resistant mutant of *Enterococcus faecalis* was found to be less hydrophobic than the wild type parent strain [48]. Partitioning of cells in the non-polar solvent hexadecane was measured to determine whether modulating cell hydrophobicity was a *S. aureus* response to growth in the presence of fatty acids. Growth in the presence of 0.1 mM linoleic acid resulted in both strains exhibiting decreased partitioning indicating a decrease in cell surface hydrophobicity (Fig. 3C). The change in cell hydrophobicity was particularly dramatic for MRSA252 with partitioning reduced from over 90% to less than 20% of cells upon growth in the presence of linoleic acid. The adaptive decrease in cell hydrophobicity makes conditions less favourable for interactions between the cell and the amphipathic fatty acid. Alterations to cell surface charge via the *dlt* and *mprF* loci have also been linked to *S. aureus* evasion of a number of innate immune system components including cationic antimicrobial peptides [49]–[51]. The SH1000 mutants *vraE*, *sasF*, or *SAR2632*, identified in this study as having decreased survival upon exposure to linoleic acid, did not exhibit altered hydrophobicity in this partitioning assay (data not shown). This indicates that the products of these three genes interact with LC-uFFAs in a manner that does not involve alterations to cell surface hydrophobicity.

### Murine arthritis virulence assay

**Figure 4:**
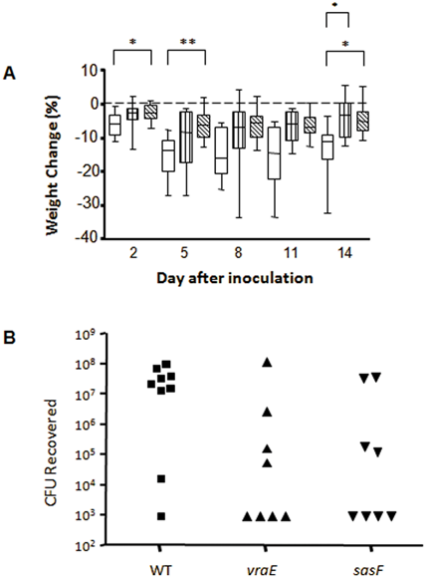
Contribution of *vraE* and *sasF* to virulence. A Effect of WT SH1000 (open box) and mutations of *vraE* (vertical hatched box) and *sasF* (diagonal hatched box) on percentage change in weight of infected mice. *p<0.05, **p<0.01 by Dunn's test. B Effect of mutations of *vraE* (closed triangle) and *sasF* (closed inverted triangle) on cfu of *S. aureu*s SH1000 (closed box) in kidneys of infected mice.

A murine arthritis model of infection was used to determine a role for the LC-uFFA survival genes *sasF* and *vraE* in pathogenesis. This model of infection also reports on systemic inflammation and abscess formation in kidneys and was therefore relevant for *in vivo* investigation of fatty acid survival mutants. Neither the *sasF* nor *vraE* mutations showed a significant reduction in arthritis development of SH1000 in this model (data not shown). However, a significantly reduced weight loss (P<0.05) was observed for both *sasF* and the *vraE* mutants for 3 out of 5 weight measurements over the 14 day experiment, when compared to the SH1000 parent strain (Fig. 4A). In contrast, while a reduced bacterial load of both mutant strains was observed in the kidney compared to the wild type this was not found to be significant (p = 0.075) (Fig. 4B). Collectively, these data suggest that SasF and VraE might make contributions to the pathogenesis of systemic inflamation, but not to the development of arthritis.

## Discussion

Analysing the response of MRSA252, an EMRSA-16 clone, to the LC-uFFAs linoleic [C18:2 (n-6,9)] and oleic [C18:1 (n-6)] acid revealed modulated expression of many genes, including those encoding virulence determinants. After exposure of exponentially growing cells to linoleic acid there was a very large increase in RNAIII compared to control cells, and this was also observed at all stages of growth when either linoleic or oleic acid were present from the time of inoculation. This observed up-regulation of RNAIII synthesis was unexpected given previous reports on the effects of GML, a lauric acid monoester, and the LC-uFFA hexadecenoic acid [C16:1 (n-6)] on *S. aureus* gene expression [34], [35]. In those studies, there was no change in *agr* (RNAIII) expression, but down-regulation of *agr*-regulated virulence determinants, including alpha toxin (*hla*). MRSA252 has a nonsense mutation in *hla*, which does not affect *hla* mRNA measurements by qRT-PCR but ablates activity of the encoded protein preventing activity measurements [52]. In this study, transcription of *hla* in MRSA252 was only up-regulated in the presence of linoleic or oleic acid in the post-exponential growth phase demonstrating maintenance of its temporal expression, despite up-regulation of RNAIII at earlier phases of growth. Analysis by qRT-PCR revealed contrasting regulation of RNAIII synthesis in SH1000 and MRSA252 in response to treatment with LC-uFFAs. RNAIII levels were reduced after growth exposure to linoleic or oleic acid during growth of SH1000. The data reported here therefore highlights important differences between the effects of these LC-FFAs between strains. Previous studies identified a fatty acid modifying enzyme (FAME) in strains of *S. aureus*, which esterifies LC-FFAs with cholesterol, thereby reducing toxicity [32]. However, this activity was demonstrated to be *agr*-regulated [29], [53], producing the anomaly that in strains with SH1000-like regulation, expression of the detoxifying enzyme would be down-regulated upon exposure to its substrate. MRSA252 is a successful epidemic strain of *S. aureus* and the ability to persist in an environment containing LC-uFFAs such as on the skin surface (hexadecenoic acid) or in skin infections (linoleic and oleic acid) would aid the transmission of the organism. In this scenario, the specific up-regulation of *agr* in response to LC-uFFAs observed in MRSA252 (EMRSA-16) may contribute towards its success as an epidemic strain. Superior skin colonisation was previously suggested as a reason for the epidemic nature of the EMRSA-15 and -16 strains, which together are responsible for over 95% of MRSA from cases of nosocomial bacteraemia in the UK [54], [55].

Microarray analysis revealed further virulence factors exhibiting increased transcription, including the *esx* locus, which encodes a specific secretion system and the ESAT-6-like proteins that have been confirmed as having a role in the pathogenesis of murine abscesses [41]. Increased transcription of the *esx* locus was only observed after growth exposure to linoleic or oleic acid and not in response to linoleic acid challenge conditions. Increased transcription of the *arcABDC* operon, encoding the arginine deiminase (ADI) pathway enzymes, was observed under the same conditions where the *esx* locus is up-regulated. The ADI pathway enables the utilisation of arginine as an energy source under anaerobic conditions of growth. Concomitant with the expression of the ADI pathway, there was an up-regulation of many glycolytic enzymes, suggesting that a net effect of growth exposure to linoleic acid was metabolic alterations leading toward anaerobic growth. To test the importance of the ADI pathway under these conditions, an *arcA* allelic replacement mutant of SH1000 was generated (*arcA* was transcriptionally up-regulated in both SH1000 and MRSA252). The *arcA* strain was found to display a reduction in growth on agar plates containing 1 mM linoleic acid, with a 25-fold lower survival than the parental strain. The alteration in metabolism via up-regulation of the ADI pathway is therefore important for survival under these conditions. The ADI pathway may also contribute to virulence since some ST8-SCCmecIVa (USA300) MRSA clones carry the arginine catabolism mobile element (ACME), which contains an extra copy of the *arc* operon [56]. This leads to the hypothesis that the *arcABDC* operon facilitates pathogenicity by increasing survival of *S. aureus* in the presence of LC-uFFAs.

The *sasF* gene showed the largest change in expression of any gene in response to linoleic acid challenge (>16-fold and >30-fold up-regulation in MRSA252 by microarray and qRT-PCR, respectively). Expression of SasF, an LPXAG motif cell wall-anchored surface protein, is repressed by TcaR, the teicoplanin-associated locus regulator [42], [57]. The *tcaR* gene was found by microarray analysis to be up-regulated (>3-fold) in MRSA252 under the linoleic challenge conditions (Table 1). However, the SH1000 strain harbours a truncated copy of *tcaR* [42], [58] and synthesises a non-functional protein. This could explain why the *sasF* gene was only slightly up-regulated in SH1000 since its transcription may already be very high as its expression is reduced as part of the TcaR regulon. Many of the differences observed in the transcriptional responses of SH1000 and MRSA252 to the presence of fatty acids (Table 7, 8) are thus likely to be due to differential responses modulating *RNAIII* production, altered *sarA* transcription and differences between the strains in respect of the functionality of TcaR. The importance of *sasF* transcription for adaptation and survival of *S. aureus* to linoleic acid was tested by constructing an allelic replacement mutant in SH1000. The *sasF* mutant showed much reduced survival on agar plates containing 1 mM linoleic acid, exhibiting a 6-fold lower level of survival than the parental strain (Fig. 2A). The expression of this cell wall-anchored protein is therefore important for survival under these conditions. SasF may also contribute to virulence since in a murine arthritis model of infection a *sasF* allelic replacement mutant of SH1000 caused significantly less weight loss of the animals compared to control cells (Fig. 4A). Reduced numbers of bacteria were harvested from the kidney in mice infected with the *sasF* mutant strain compared to the control but the difference was not significant (Fig. 4B). SasF did not significantly affect development or severity of arthritis.

A screen for additional mutants of SH1000 that were defective for survival in the presence of linoleic acid identified several Tn*917* transposants from a 5,000 clone library of mutants. Sequencing located the transposons within genes encoding VraE (ABC transporter permease) and SAR2632 (MMPL domain, putative efflux pump). The mutants Liv753 (*vraE*) and Liv766 (*SAR2632*) had reduced survival using this agar plate-based assay exhibiting 130-fold and 4-fold reductions in viability, respectively, at 1 mM linoleic acid (Fig. 2A). Each of these genes encodes transporter proteins of unknown function. The gene *vraE* is located downstream of *vraD* in a bicistronic operon and is a member of the GraSR regulon proposed to regulate traffic of cell wall substrates [59], [60]. Two studies have shown that *S. aureus vraE* mutants display decreased resistance to meticillin and increased susceptibility to human β-defensin 3. [61], [62]. VraE may also contribute to virulence, since in a murine arthritis model of infection a *vraE* allelic replacement mutant of SH1000 resulted in significantly less weight loss of the animals compared to control cells (Fig. 4A). Reduced numbers of bacteria were harvested from the kidney in mice infected with the *vraE* mutant strain compared to control but the difference was not significant (Fig. 4B). VraE did not significantly affect the development or severity of arthritis (data not shown). SAR2632 is a predicted transporter protein of the MMPL domain family proposed to be involved in lipid transport [63].

**Figure 5:**
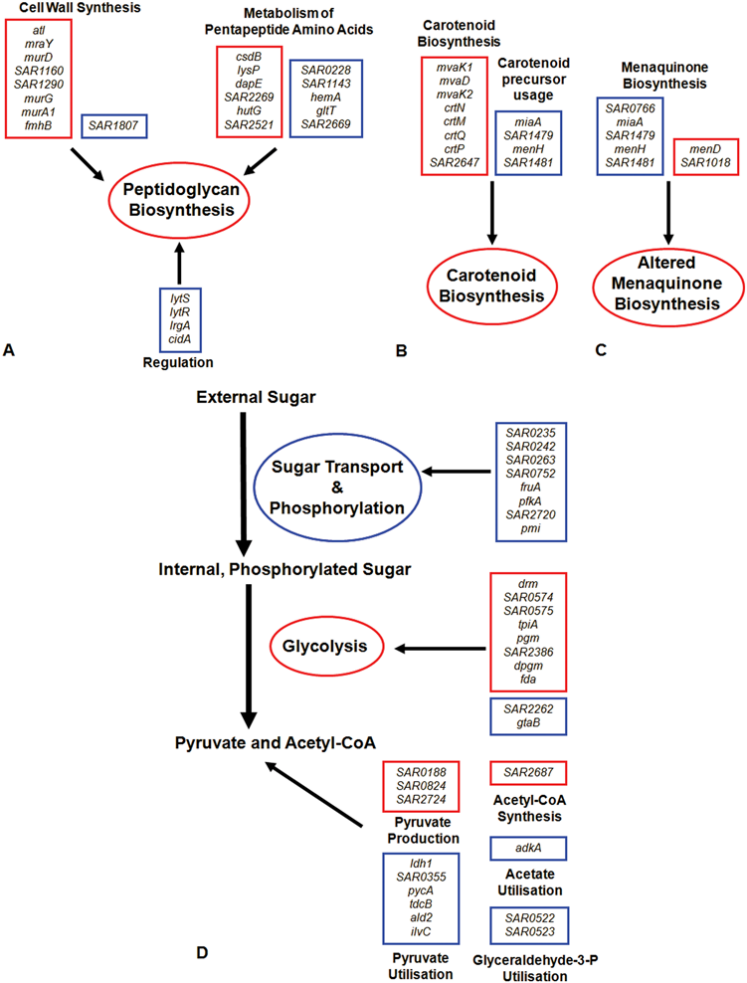
Schematic representation of cellular pathways displaying changes in gene transcription in response to linoleic acid challenge conditions. Sections A, B, C and D highlight the various genes involved in peptidoglycan, carotenoid, menaquinone and energy metabolism respectively. Genes in red and blue boxes are up- and down-regulated, respectively. See text for details.

The identification of cell envelope mutants correlated with the gene expression and proteomic data, in which altered levels of cell wall synthesis and regulation components was observed (Fig. 5A). An increase in autolysis was observed under growth exposure conditions, although whether this is due to changes in expression of PG synthesis genes or down-regulation of the CtsR regulon remains unelucidated. The overall up-regulation of many cell wall synthesis genes could have two possible explanations. Firstly, the increased synthesis may be required to maintain the integrity of the cell wall, damaged due to loss of material through the precipitation of PG by LC-uFFAs as described by Campbell *et al*. [25]. The binding of LC-uFFAs to PG would not be unexpected given that chitosan, which has a very similar structure to PG, has been shown to bind lipids [64]. Secondly, an increase in cell wall and capsule synthesis could act as a defense mechanism since an increase in ionically charged material surrounding the cell would mitigate against access of the non-polar carbon chain of LC-uFFAs to the cell membrane. Reduced cell surface hydrophobicity and increased thickness of the cell wall have been suggested as Gram-positive defense mechanisms to limit interactions with lipids [34], [48]. Therefore, the reduced cell surface hydrophobicity of both the MRSA252 and SH1000 strains, observed here following overnight growth in the presence of linoleic acid, represents a pathogen countermeasure to this component of the innate immune system.

Previous studies have used gene expression profiling to determine the cellular pathway targeted by antimicrobial agents [65], [66]. In this study, there was no change in expression of fatty acid biosynthesis genes, other than *fabZ*, which is located on the same operon as the PG synthesis gene *murA1*. This suggests that the anti-staphylococcal toxicities of the LC-uFFAs used in this study are not a consequence of inhibiting fatty acid biosynthesis. Prior studies on the action of LC-uFFAs upon cells of *S. aureus* demonstrated membrane perturbations [12], [18], [22], [26]. This supports the finding of Chamberlain *et al.* that increased fluidity of *S. aureus* membranes resulted from exposure to LC-uFFAs [26]. Furthermore, these authors demonstrated that carotenoid-dependent pigmentation in non-isogenic clinical isolates positively correlated with increased survival from LC-uFFAs and showed that the carotenoid staphyloxanthin acted to decrease membrane fluidity and reduce its damaging effects. Interestingly, genes involved in carotenoid biosynthesis were up-regulated in response to LC-uFFAs (Fig. 5B), and Liv681 (*crtM*), which cannot produce staphyloxanthin, was shown here to have a >5-fold reduced survival to 1 mM linoleic acid. Staphyloxanthin production is regulated via σ^B^, which also regulates many stress response components observed to be up-regulated in the array data. Consequently, the general stress response, including the production of staphyloxanthin serves as an important component of defence against LC-uFFAs, given the discovery that Liv130 (*sigB*) exhibited a >75-fold reduction in survival to 1 mM linoleic acid. *sigB* has previously been shown to contribute to *S. aureus* resistance to antimicrobials [67]. The CtsR regulon was strongly up-regulated after linoleic acid challenge, but was down-regulated after growth exposure and may therefore also participate in the adaptation to this environment.

A consequence of LC-uFFAs inserting in the cell membrane could be the disruption of the electron transport chain. This would explain the numerous changes in expression of genes associated with energy creation within the cell (Fig. 5C, 5D) and appears to constitute the main cellular response to LC-uFFAs. The overall trend is one of increasing levels of pyruvate and alterations in menaquinone synthesis. Moreover, the ADI pathway for anaerobic utilisation of arginine was up-regulated under growth exposure conditions. The genes affected by LC-uFFAs include those involved in the glycolytic and fermentative pathways, cell wall synthesis, cell division, and capsule synthesis. These pathways have also been shown to be modulated in a *S. aureus* small colony variant, which has a mutation in the *hemB* gene of the electron transport chain [68]. This supports a mode of action for LC-uFFAs of disturbing cell energetics via membrane disruption. Finally, the overall similarities in responses to the LC-uFFAs employed in this study appear to indicate a common mode of action amongst the linoleic, oleic and hexadecenoic acids. This corroborated dataset on the transcriptional and translational responses of *S. aureus* should provide a useful resource for further studies on this pathogens response to the host environment.

## Materials and Methods

### Bacterial strains, plasmid and growth conditions

Strains and plasmids used in this study are listed in Table 13. Unless indicated otherwise, bacteria were grown in brain heart infusion broth (BHI)(Merck) at 37°C with shaking at 125 rpm. When included, antibiotics were added at the following concentrations: erythromycin, 5 µg ml^−1^; lincomycin, 25 µg ml^−1^; tetracycline, 5 µg ml^−1^, chloramphenicol 10 µg ml^−1^.

**Table 13:** Strains and plasmids used in this study.

Strain or Plasmid	Comment	Reference or Source
Strains:		
*E. coli*:		
DH5α	*ø80* (*lacZ*)*M15* (*argF-lac*)*U169 endA1 recA1 hsdR17* (*r_K_^−^ m_K_^+^*) *deoR thi-1 supE44 gyrA96 relA1*	[86]
*S. aureus*:		
SH1000	Functional *rsbU* derivative of 8325-4 *rsbU* ^+^	[87]
MRSA252	Wild-type clinical isolate	[52]
RN4220	Restriction-deficient transformation recipient mutant of 8325-4	[88]
N315	Wild-type pharyngeal smear clinical isolate	[89]
MSSA476	Wild-type clinical isolate	[52]
Liv033 (*katA*)	8325-4 *katA::*Tn*917*	[87]
Liv038 (*agr*)	SH1000 *agr::tet*	[87]
Liv039 (*sarA)*	SH1000 *sarA::kan*	[87]
Liv101 (*lytS*)	SH1000 *lytS::*pER1	[45]
Liv130 (*sigB*)	SH1000 *sigB::tet*	[87]
Liv142 (*atl*)	SH1000 *atl::lacZ* pAZ106	[44]
Liv405 (*clfA*)	8325-4 *clfA::lacZ* pAZ106	T. Foster
Liv442 (*clfA*)	SH1000 *clfA* trandsduced from Liv405	This Study
Liv671 (*clpC*)	SH1000 *clpC::erm*	[90]
Liv673 (*crtM*)	Newman *crtM::cat*	[91]
Liv681 *(crtM*)	SH1000 *crtM::cat* transduced from Liv673	This study
Liv684 (*sasF*)	RN4220 *sasF::tet*	This Study
Liv686 (*arcA*)	RN4220 *arcA::tet*	This Study
Liv692 (*arcA*)	SH1000 *arcA::tet* transduced from Liv686	This Study
Liv694 (*sasF*)	SH1000 *sasF::tet* transduced from Liv684	This Study
Liv718 (*vraS*)	SH1000 *vraS::tet* transduced from Liv723	This Study
Liv723 (*vraS*)	RN4220 *vraS::tet*	This Study
Liv750 (*katA*)	SH1000 *katA::*Tn*917* transduced from Liv033	This study
Liv753 (*vraE*)	SH1000 *SAR2782::*Tn*917*	This Study
Liv766 (*SAR2632*)	SH1000 *SAR2632::*Tn*917*	This Study
Liv994	RN4220 pSK5630+*sasF*	This Study
Liv995	RN4220 pSK5630+*arcA*	This Study
Liv996	RN4220 pSK5630+*vraE*	This Study
Liv997	RN4220 pSK5630+*SAR2632*	This Study
Liv1000 (*sasF*)	Liv694 complemented with pSK5630+*sasF*	This Study
Liv1001 (*arcA*)	Liv692 complemented with pSK5630+*arcA*	This Study
Liv1002 (*vraE*)	Liv753 complemented with pSK5630+*vraE*	This Study
Liv1003 (*SAR2632*)	Liv766 complemented with pSK5630+*SAR2632*	This Study
Plasmids:		
pLTV1	Temperature-sensitive plasmid harbouring Tn*917*	[69]
pAZ106	Promoterless *lacZ erm* insertion vector	[92]
pDG1513	pMTL22 derivative [tet^r^]	[73]
pSK5630	Low copy number *E. coli-S. aureus* shuttle vector [cm^r^]	[75]

### Transposon mutagenesis and screening for sensitivity to LC-uFFAs

Transposon mutagenesis was performed on the SH1000 strain of *S. aureus* using the Tn*917* containing plasmid pLTV1, as described previously [69]. Single colonies from a transposon library grown on BHI agar containing erythromycin and lincomycin were innoculated into 96-well plates containing 200 µl of BHI. From this library 5,000 clones were cultured and stored at −80°C in 10% glycerol. After repeat growth of clones overnight at 37°C, without shaking, cultures were diluted 100-fold before 5 µl was spotted onto BHI agar with or without 0.5 mM linoleic acid. After overnight incubation, strains with decreased resistance to linoleic acid, when compared to wild type (WT) SH1000, were selected. The transposon-mediated mutations in these strains were transduced into the WT SH1000 using ø11 as described previously [70]. The linoleic acid sensitivity of these mutants was reconfirmed, proving the phenotype was transposon-linked, by repeat assay using serial dilutions of the mutant strains onto BHI agar containing millimolar concentrations of linoleic acid. The locations of the Tn*917* insertions within the genome of mutants were determined using arbitrary primed nested PCR and DNA sequencing of regions upstream and downstream of the transposon [71].

### Construction of *sasF*, *arcA* and *vraS* insertional mutants and complementation plasmids

Construction of *sasF, arcA* and *vraS* mutants was performed as described by Horsburgh *et al*. [72] using the oligonucleotides described in Table 14. Briefly this was as follows: the *sasF*, *arcA* or *vraS* genes were amplified as upstream and downstream fragments using primer pairs sasF_*BamH*I/sasF_*Not*I and sasF_*Kpn*I/sasF_*EcoR*I, or arcA_*BamH*I/arcA_*Not*I and arcA_*Kpn*I/arcA_*EcoR*I or vraS_*BamH*I/vraS_*Not*I and vraS_*Kpn*I/vraS_*EcoR*I, respectively. The tetracycline resistance gene from pDG1513 [73] was amplified by using the primer pair Tet_*Not*I/Tet_*Kpn*I. The upstream, downstream and *tet* gene fragments were digested with *BamH*I and *Not*I, or *Kpn*I and *EcoR*I, or *Not*I and *Kpn*I, respectively, and simultaneously ligated into pAZ106, which had been previously digested with *BamH*I and *EcoR*I. The resulting constructs were confirmed by restriction digest and then used to transform electrocompetent *S. aureus* RN4220 by the method of Schenk and Ladagga [74]. Strains of RN4220 containing the Campbell integration of the plasmid were resolved in SH1000 by transductional outcross using ø11. Clones of SH1000, which had now lost the plasmid and contained an allelic replacement with the tetracycline resistance gene, were confirmed as mutants by PCR amplification. Correct allelic replacement was confirmed in each case.

**Table 14:** Oligonucleotides used for the construction of mutants.

Oligonucleotides	Sequence (5′ to 3′)
sasF_BamHI	CCACGGATCCGGTAGTGATGTTTTGG
sasF_NotI	ATAACTGCGGCCGCTTGAAACGGTTTCCCTCG
sasF_KpnI	CCGGTACCGTTATCACGACGCAATAAG
sasF_EcoRI	ACATGAATTCAAACAAGGAGTTCGGAC
arcA_BamHI	CCACGGATCCACAAGTAGTAGATATGTG
arcA_NotI	ATAACTGCGGCCGCTTAATTGGACCATCTGTC
arcA_KpnI	CCGGTACCGACACTTTCTAATCAAG
arcA_EcoRI	ACATGAATTCTGCTTTGGTAAATCAC
vraS_BamHI	CCACGGATCCGCATGCTAGCTGCATTTC
vraS_NotI	ATAACTGCGGCCGCCATTTCATGATCATCCAC
vraS_KpnI	CCGGTACCCAAGCTGTCATCTATGCATTC
vraS_EcoRI	ACATGAATTCGCTGAAACATCTACTC
Tet_NotI	ATAACTGCGGCCGCGGCGGATTTTATGACCGATGAAG
Tet_KpnI	CCGGTACCTTAGAAATCCCTTTGAGAATGTTT
Complementation	
Sar2725_SasF_For	ACGCGTCGACTAATATGATGTTAGCGACATGG
Sar2725_SasF_Rev	ACGCGGATCCAATGATGGACAATCTATTCATTGC
arcA_For	ACGCGTCGACGTGAATATAATCACATGTAAGCG
arcA_Rev	ACGCGGATCCTCTGTCATTATTTTCACCCTCG
Sar2632_For	ACGCGTCGACTTTATAACTCGTAAATCAGTCTC
Sar2632_Rev	ACGCGGATCCCATGTAAAATTTGCGACATTGC
Sar2782_vraE_For	ACGCGTCGACTGTCATCATGCTAAAAGATGGC
Sar2782_vraE_Rev	ACGCGGATCCAGTTAATAGTTATACTGCATTGC

Complementation of the *sasF, arcA, vraE* and *SAR2632* mutants was performed by amplifying each gene with sufficient upstream and downstream DNA using the primer pairs listed in Table 14. The fragments were ligated into pSK5630 [75] following digestion with *BamH*I/*Sal*I, and the resulting constructs and the control plasmid were transformed into *E. coli* DH5α, with selection on agar plates containing ampicillin. The resulting constructs were confirmed by restriction digest and then used to transform electrocompetent *S. aureus* RN4220. The plasmids were then purified from RN4220 and transformed into the corresponding mutant strains.

### Microarray analysis

To ascertain the transcriptional responses of MRSA252 to fatty acids, overnight cultures (18 h) of MRSA252 were used to inoculate 100 ml of BHI (Merck) with or without 10 µM of oleic or linoleic acid in 250 ml conical flasks. These 100 ml cultures were placed in a shaking water bath at 37°C at 250 rpm and 10 ml samples were taken from the flask when the cultures reached late exponential phase (OD_600_ = 3). Identical inoculations were performed to 100 ml of BHI lacking additional fatty acids. 100 µM of linoleic acid in ethanol or an equal volume of the ethanol used to dilute the fatty acid was added to these cultures at an OD_600_ = 3.0 and the RNA extracted from treated and untreated cells 20 min post-treatment. Each treatment and control culture was performed in biological triplicate. The concentrations of fatty acids used in these experiments did not alter the pH of the media. RNA was extracted from 10 ml samples of culture taken at the indicated time intervals and stabilised by the addition of 20 ml of RNA Bacteria Protect (Qiagen). The cells were subsequently harvested by centrifugation at 5000 rpm for 10 min and cell pellets resuspended in lysis buffer (10 mM Tris, pH8.0) containing 200 U ml^−1^ of lysostaphin and 400 U ml^−1^ of mutanolysin, and incubated for 90 min at 37°C with gentle shaking every 10–15 min. The RNA was subsequently extracted using the RNeasy Midi kit (Qiagen) and DNase treated whilst on the purification column using the RNase-Free DNase Set according to manufacturers instructions (Qiagen). The quantity and quality of the RNA was assessed on an Agilent 2100 bioanalyzer by using the RNA 6000 Nano LabChip Kit. The RNA was converted to cDNA and labelled by incorporation of Cy5 dCTP during reverse transcription of RNA using the enzyme Superscript II (Amersham). DNA used in the microarray hybridisations was extracted from 5 ml of an overnight culture (18 h) of MRSA252 using the Edge Biosystems Bacterial Genomic DNA purification kit according to manufacturer's instructions. The DNA was labeled by the incorporation of Cy3 dCTP using Klenow (Invitrogen). cDNA derived from RNA and genomic DNA were pooled and hybridized on whole-genome microarrays supplied by the Bacterial Microarray Group at St. George's Hospital (BμG@S [http://bugs.sgul.ac.uk]) before washing and scanning [76]. Microarrays were scanned using an Affymetrix 428 scanner and image data extracted using ImaGene 5.2 (BioDiscovery). Fully annotated microarray data have been deposited in BμG@Sbase (accession number E-BUGS-68; http://bugs.sgul.ac.uk/E-BUGS-68) and also ArrayExpress (accession number E-BUGS-68). Two independent labelling reactions and hybridisations were carried out for each RNA sample. Image data was analysed using the GeneSpring 7.3.1 software (Silicon Genetics). Briefly, data were normalized relative to the corresponding untreated controls. Signals below 0.01 were taken as 0.01. Genes were then filtered on expression level to remove non-changing genes, with only those genes that changed by at least two-fold considered biologically significant. Changing genes were then filtered on confidence applying the Benjamini and Hochberg false discovery rate algorithm with a maximum significance cut-off at 0.05 to eliminate the chance of false-positives [77].

### Quantitative Real-Time PCR

To confirm the validity of microarray data gene specific mRNAs were quantified from treated and untreated cultures by quantitative real-time PCR (qRT-PCR). Cells were grown in biological triplicate exactly as described above for the microarray experiments and bacterial RNA was isolated using the Pro-Blue Fast RNA kit (MP Biomedicals). DNA was removed from the samples by DNase I treatment (Ambion) according to manufacturer's instructions. The purified RNA was quantified using the nanodrop ND-1000 Spectrophotometer (Thermo Fisher Scientific) and the integrity assessed by electrophoresis. 0.5 µg of RNA was reverse transcribed with 100 U of Bioscript Reverse Transcriptase (Bioline) using 0.2 µg of random hexamer primers (Promega) according to manufacturer's instructions. qRT-PCR was performed using the 7500 Fast System (Applied Biosystems) and the QuantiFast SYBR Green PCR kit (Qiagen) according to manufacturer's instructions. The relative levels of gene expression in fatty acid treated cells and the non-treated controls were calculated by relative quantification using *gyrB* as the endogenous reference gene. The choice of *gyrB* as a single reference gene was based on its consistent levels in microarray in all conditions and at all timpoints that were analysed. The oligonucleotides used for qRT-PCR are listed in Table 15. All samples were amplified in triplicate and the data analysis was carried out using the 7500 Fast System Software (Applied Biosystems).

**Table 15:** Oligonucleotides used for qRT-PCR analysis.

Oligonucleotide	SAR Number	Sequence (5′ to 3′)
gyrB_For	*SAR0005*	ATCGACTTCAGAGAGAGGTTTG
gyrB_Rev	*SAR0005*	CCGTTATCCGTTACTTTAATCCA
spa_For	*SAR0114*	GAAGCAACCAGCAAACCATGC
spa_Rev	*SAR0114*	ACGTCCAGCTAATAACGCTGC
fadA_For	*SAR0223*	GAAGATGTCATTGTTGGTACGG
fadA_Rev	*SAR0223*	TGTAATCCTGATGAGCAGTAGC
fadD_For	*SAR0225*	TTCATTGCTAGAAAGTAAGTACCG
fadD_Rev	*SAR0225*	TGGCGTTTGGACGATCCTTGT
lytR_For	*SAR0258*	TTTTTGCAACTGCACATGACCAA
lytR_Rev	*SAR0258*	TTATCATCTTTGGCTTTAGTCGC
sarA_For	*SAR0625*	TAAACTACAAACAACCACAAGTTG
sarA_Rev	*SAR0625*	TTCGATTTTTTTACGTTGTTGTGC
clpB_For	*SAR0938*	GAACGAGCAAATATTGAGGTAGA
clpB_Rev	*SAR0938*	GCCTTAGTTATCAATTGGTTTGC
fabI_For	*SAR0978*	GTGATGGGTGTTGCTAAAGCG
fabI_Rev	*SAR0978*	AACCACCCACACCTTTTGCAC
hla_For	*SAR1136*	GTTGCAACTACCTGATAATGAAG
hla_Rev	*SAR1136*	CCAATTTTTCCAGAATCATCACC
katA_For	*SAR1344*	AATAGTATGACAGCAGGGCCTA
katA_Rev	*SAR1344*	AATGTCCCAAATGCACCAGAAC
murG_For	*SAR1430*	ATCCCGAGGCGACCAAATTGA
murG_Rev	*SAR1430*	AATTCGAGTTCTTTCCTGTTCCA
fabZ_For	*SAR2186*	AATATGAAGAAGGTCAACGTTGC
fabZ_Rev	*SAR2186*	ACCGCACCTGTTTGAGCTAACG
cidA_For	*SAR2621*	GCCGGCAGTATTGTTGGTCTA
cidA_Rev	*SAR2621*	TAATACCTACAACTGACGGTATG
crtM_For	*SAR2643*	TGATGACAGTATAGATGTTTATGG
crtM_Rev	*SAR2643*	ACATGCTGAAGGGCCATCATG
arcA_For	*SAR2714*	GTCAGGAGTACGTAAGGAAGA
arcA_Rev	*SAR2714*	GTGTCCTATTGAGGCTTGTGG
sasF-For	*SAR2725*	CACAAATCGGAAGATTCAGC
sasF_Rev	*SAR2725*	TGAGTCGATTACTATGGCTTTGA
RNAIII_For	RNAIII	ACATGGTTATTAAGTTGGGATGG
RNAIII_Rev	RNAIII	TAAAATGGATTATCGACACAGTGA

### Sample preparation for 2D-PAGE

Cultures of MRSA252 (100 ml) were grown to late exponential phase (OD_600_ = 3.0) and exposed to 0.1 mM linoleic acid or 0.1 mM hexadecenoic acid as described above. Cells were harvested by centrifugation at 5000 g for 10 min at 4°C. After two washes in PBS the cells were resuspended in 2 ml of lysis buffer (PBS, 1 mg/ml DNase I, 100 µM benzamidine, 100 µM PMSF, 1 mg/ml RNase, 2 mg/ml lysostaphin) and incubated at 37°C for 20 min before chilling on ice. Cell debris and insoluble material was pelleted by centrifugation at 4°C for 20,000 g for 20 min. The supernatant was stored at −20°C. Protein samples were quantified using the BioRad Protein assay. The protein samples were desalted using Slide-A-Lyzer Mini Dialysis Units with a 3.5 kDa MWCO (Thermo Scientific).

### 2D-PAGE

Soluble protein (120 µg ) was brought up to 320 µl with rehydration buffer (8 M urea, 2M thiourea, 4% (w/v) CHAPS, 20 mM DTT, 1% (v/v) ASB 14 detergent and 0.5% (v/v) carrier ampholytes (Bio-lyte 3/10, Bio-Rad)). Samples were incubated for an hour at room temperature with gentle shaking, before centrifugation at 8,000 g for 5 min. Samples were in-gel rehydrated and focused on 17 cm, pH 4–7 IPG strips (Bio-Rad) for a total of 40000 V h (150V for 1h, 300V for 1h, 600V for 1h, 1200V for 1h, 1200–8000V over 1h (linear gradient), 8000 V to 40000 v (steady state)), using a Protean IEF Cell (Bio-Rad). After focusing, strips were equilibrated in 50 mM Tris (pH 6.8), 6 M urea, 2% (w/v) SDS, 30% (w/v) glycerol, and bromophenol blue, containing 20 mM DTT in the reduction step (15 min) and 25 mM iodoacetamide in the alkylation step (15 min). IPG strips were run in the second dimension on 20×20cm 12.5% SDS-PAGE gels using a Protean II xi 2D Cell (Bio-Rad). Gels were run in triplicate, silver-stained [78] and scanned (GS-710 Densitometer, Bio-Rad) as gray-scale tiff files at 16 bit and 300 dpi and uploaded into the Progenesis ‘SameSpots’ (Non Linear Dynamics) gel image analysis Software. Quantitative analysis was based on average gels created from three gel replicates. Spots in the treated samples with a p≤0.05 and ≥ two-fold difference from the control sample were considered statistically significant. For protein identification by mass spectrometry 2 gels containing 800 µg each of soluble protein (a pool from each growth condition) were prepared as above and stained with Colloidal Coomassie Brilliant Blue [79]. The scanned images were uploaded into Progenesis ‘SameSpots’ and matched to the analytical gels.

### Trypsin digestion and mass spectrometric identification of proteins

Spots for identification were excised and digested in-gel with trypsin. Gel Plugs were destained in 50% (v/v) acetonitrile:50% (v/v) 50 mM ammonium bicarbonate (37°C), dehydrated in 100% acetonitrile (37°C), and rehydrated overnight (37°C) in 10 µl of 50mM ammonium bicarbonate containing trypsin (1 µl of 100 ng trypsin stock reconstituted in 50 mM acetic acid (Promega)). Supernatants containing the extracted peptides were removed and analyzed by MALDI-TOF.

Peptide Mass Fingerprinting (PMF) was conducted on a reflectron MALDI-TOF instrument (MLDIWatersMicromass,UK ). Samples were mixed in a 1∶1 ratio with a saturated solution of α cyano-4-hydroxycinnamic acid ACN: water:TFA (50∶49∶1 (v/v/v)). The acquired spectra were analysed using MassLynx v 4.0 (Waters-Micromass,UK) and were all externally calibrated with a mixture of peptides. For each sample, all acquired spectra were combined and processed as follows using MassLynx v 4.0: smoothing, 2× smooth using a Savitzky Golay method set at +/− 3 channels and background subtraction using a polynomial of order 1 and 40% below the curve in order to reduce background noise. To get accurate mono isotopic peak data all processed spectra were centred using the top 80% of each peak. Peak lists were generated using ProteinLynx, part of MassLynx v 4.0. Monoisotopic peptide masses in the mass range of 800–4000 Da were used in the database search. The resulting peptide mass maps were used to interrogate S. *aureus* MRSA252 sequences to generate statistically significant candidate identifications using the MASCOT search engine (Matrix Science). Searches were performed allowing for complete carbamidomethylation (alkylation) of cysteine residues, partial oxidation of methionine residues, one missed cleavage and a mass error of 250 ppm. Molecular Weight Search (MOWSE) scores [80], number of matched ions, percent protein sequence coverage, and correlation of gel region with predicted mass and pI were collectively considered for each protein identification.

### Cell surface hydrophobicity assays

Cell surface hydrophobicity of *S. aureus strains* was measured as described previously [81]. Briefly, stationary-phase cells (18-h cultures) grown in the presence or absence of 0.1 mM linoleic acid were harvested, washed three times and resuspended to an OD_440_ of 0.5 in PBS. 3 ml aliquots of each of these bacterial suspensions were vortexed for 1 min with 200 µl *n*-hexadecane (Sigma). After 15 min incubation to enable partitioning, 1 ml was removed from the aqueous layer and the OD_440_ recorded. Cell surface hydrophobicity was calculated as the percentage decrease in OD as a result of cells partitioning into the hexadecane.

### Cell Autolysis Assay

Cell autolysis rates were determined on cells exposed to linoleic acid using an assay modified from that described by Blackman *et al*. [82]. Briefly, cells were grown in 100 ml volumes of BHI to an OD_600_ of 0.8–1.0 in the presence or absence of 0.01 mM linoleic acid. Following harvesting of the cells by centrifugation, the cells were washed in PBS and resuspended to an OD_600_ = 0.6 in 0.5% (v/v) Triton X-100. The cells were incubated with shaking at 37°C and the OD_600_ was monitored over time.

### Experimental septic arthritis

A well described mouse model of septic arthritis was used to test the *in vivo* role of genes implicated in resistance to fatty acids in the strains [83]–[85]. Seven week old female NMRI mice were obtained from Charles River Laboratories (Sulzfeld, Germany) and maintained in the animal facility of the Department of Rheumatology and Inflammation Research, University of Göteborg, Sweden. All mice were maintained according to the local ethic board animal husbandry standards. The mice were housed 10 to a cage under standard conditions of temperature and light and were fed standard laboratory chow and water *ad libitum*. Bacteria were grown on blood agar plates for 24 h, harvested and stored frozen at −20°C in PBS containing 5% bovine serum albumin and 10% dimethyl sulfoxide. Before injection into animals, the bacterial suspensions were thawed, washed in PBS, and adjusted to appropriate cell concentrations. Mice were inoculated in the tail vein with 0.2 ml of bacterial suspension. The number of viable bacteria was measured in conjunction with each challenge by counting colonies following culture at 37°C for 24 hours on blood agar plates. Ten mice were infected with each strain of *S. aureus* by i.v. injection in the tails of 3.2–3.5×10^6^ CFU of bacteria for induction of septic arthritis. The mice were weighed regularly and examined for arthritis until death by cervical dislocation 14 days after challenge. The kidneys were aseptically dissected and kept on ice, homogenized, diluted in PBS and inoculated on blood agar plates. Data were presented as CFU per kidney pair.
